# Potent Alkaline Phosphatase Inhibitors, Pyrazolo-Oxothiazolidines: Synthesis, Biological Evaluation, Molecular Docking, and Kinetic Studies

**DOI:** 10.3390/ijms232113262

**Published:** 2022-10-31

**Authors:** Narges Hosseini Nasab, Hussain Raza, Rok Su Shim, Mubashir Hassan, Andrzej Kloczkowski, Song Ja Kim

**Affiliations:** 1 Department of Biological Sciences, Kongju National University, Gongju 32588, Chungnam, Korea; 2 Department of Pediatrics, The Steve and Cindy Rasmussen Institute for Genomic Medicine at Nationwide Children’s Hospital, Columbus, OH 43205, USA

**Keywords:** pyrazolo-oxothiazolidine, alkaline phosphatase, kinetic analysis, cell viability, molecular docking

## Abstract

To develop new alkaline phosphatase inhibitors (ALP), a series of pyrazolo-oxothiazolidine derivatives were synthesized and biologically assessed, and the results showed that all of the synthesized compounds significantly inhibited ALP. Specifically, compound **7g** displayed the strongest inhibitory activity (IC_50_ = 0.045 ± 0.004 μM), which is 116-fold more active than monopotassium phosphate (IC_50_ = 5.242 ± 0.472 μM) as a standard reference. The most potent compound among the series (**7g**) was checked for its mode of binding with the enzyme and shown as non-competitively binding with the target enzyme. The antioxidant activity of these compounds was examined to investigate the radical scavenging effect. Moreover, the MTT assay method was performed to evaluate their toxic effects on the viability of MG-63 human osteosarcoma cells, and all compounds have no toxic effect on the cells at 4 μM. Computational research was also conducted to examine the binding affinity of the ligands with alkaline phosphatase, and the results revealed that all compounds showed good binding energy values within the active site of the target. Therefore, these novel pyrazolo-oxothiazolidine derivatives might be employed as promising pharmacophores for potent and selective alkaline phosphatase inhibitors.

## 1. Introduction

Alkaline phosphatases [ALP; orthophosphoric monoester phosphohydrolase (alkaline optimum) EC 3.1.3.1], which are widely distributed in nature from prokaryotes and higher eukaryotes to humans [1,2], are known as plasma membrane-bound glycoproteins [3,4]. Alkaline phosphatase belongs to a large family of dimeric enzymes that hydrolyzes various monophosphate esters at a high pH optimum with the release of inorganic phosphate [5,6]. It is often restricted to the cell surface [7,8]. ALP, as a representative mediator of biochemistry, actively participates in most biological processes, including metabolism, signal transduction, molecular transport, and the manifestation of genetic information. ALP has a catalytic function because of its structural composition, which consists of two monomers with five cysteine residues, two zinc atoms (Zn^2+^), and one magnesium atom (Mg^2+^) in each monomer [9,10,11]. The ALP monomer’s conformation is significantly affected by these metal ions, and they also indirectly adjust interactions between subunits [12]. Human ALPs can be divided into at least four isoenzymes, depending on the specificity of the tissue that will express them. Three of them are tissue-specific enzymes including placental alkaline phosphatase (PLALP or Regan isozyme), intestinal alkaline phosphatase (IALP), and germ cell ALP (GCALP or NAGAO isozyme), while the fourth is tissue nonspecific enzyme as liver/bone/kidney alkaline phosphatase (L/B/K ALP) [13]. By performing inorganic pyrophosphate (PPi) hydrolysis, L/B/K ALP maintains an optimum pyrophosphate level in bone tissues. The intestinal ALPs are present in the epithelial linings of the intestine and regulate the detoxification of bacterial lipopolysaccharides, bicarbonate secretion, and lipid intestinal absorption. Certain amino acids inhibit ALPs in a non-competitive manner. L-phenylalanine inhibits the tissue-specific isozymes (PLALP, GCALP, and IALP) 30 times more sensitively than the L/B/K ALPs, whereas the L/B/K ALPs are more sensitive to inhibition by l-homoarginine (Har) than PLALP, GCALP, and IALP. r–Phenylalanyl–glycyl–glycine (Pgg) gives strong differential inhibition between PLALP, IALP, and L/B/K ALPs. Levamisole (Leva) is a highly effective inhibitor of L/B/K ALP but has a minor inhibitory impact on the other ALPs [14]. 

**Figure 1:**
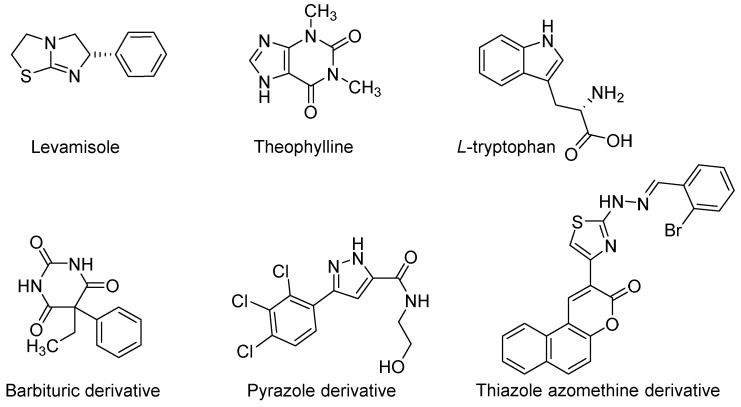
Identified inhibitors of ALP.

Numerous disorders, including bowel diseases, sepsis, arthritis, multiple sclerosis, atherosclerosis, and esophageal, breast, liver, intestinal, prostate, ovarian, and intestinal cancers are also associated with the overexpression of IALPs [15,16,17]. The elevated expression of ALP in cancer patients indicates metastasis to the liver and bone. Hepatocellular carcinomas have been discovered to have higher IALP levels [18], while osteosarcomas, breast cancer, and osteoblastic bone metastasis have all been associated with increased plasma levels of L/B/K ALP [19]. Moreover, some metabolic disorders such as Wilson’s disease or hematological diseases such as aplastic anemia and chronic myelogenous leukemia have occurred when ALP concentration is at its low level [20]. The investigation for efficient and selective ALP isozyme inhibitors is becoming more popular. In recent years, various ALP inhibitors have been reported based on biaryl sulfonamide, chromone, triazole, and coumarin sulfonate motifs [21]. Some compounds were discovered to be efficient ALP inhibitors; however, the majority of them inhibited ALPs non-selectively [22,23,24]. Traditionally, the only ALP inhibitors available were levamisole and theophylline, with K_i_ values of 16 and 82 µM, respectively [25]. However, in order to comprehend the role of ALPs in health and disease, it is imperative to develop small drug-like molecules as efficient and selective inhibitors of ALP isozymes. Figure 1 demonstrates some identified representative ALP inhibitors [26,27]. As a result, ALPs represent an attractive molecular target for drug discovery, and the development of effective and selective ALP isozyme inhibitors is desperately required.

Heterocyclic compounds have a cyclic structure in which the ring contains two or more different types of atoms. Also, a large number of heterocyclic compounds are known, and the number is continually growing. Heterocycles are well-studied bioactive compounds and thought to be attractive synthetic targets for the creation of new therapeutics [28]. One important topic for organic chemists is the synthesis of various heterocyclic compounds from straightforward and easily accessible starting materials [29,30,31,32].

Pyrazoles are a five-membered aromatic heterocyclic system from the azole branch with two adjacent nitrogen atoms. They are a significant class of heterocyclic compounds that have a variety of chemical, biological, agrochemical, and pharmacological activities [33]. In medicinal chemistry, compounds possessing pyrazole rings have shown biological activity, including antimicrobial [34,35], antifungal [34], leishmanicidal [36,37], antiviral [35], antichagasic [38], pesticidal [39], antihyperglycemic [40], anti-inflammatory [41], and antitumoral [35] activities. Similarly, it has been established that thiazolidinones, a type of five-membered heterocyclic compounds that include sulfur at position 1, nitrogen at position 3, and a carbonyl group at position 4, have significant biological activities. Its derivatives are among the most broadly investigated moieties, and the first recognition of its natural occurrence was its existence in penicillin [42]. In several fields, including chemistry, pharmacology, biology, and materials science, they have a broad spectrum of applications [43]. Thiazolidinone have biological effects such as anticonvulsant [44], hypnotic [45], antitubercular [46], antibacterial [47], anticancer [48], and anti-inflammatory [49] properties.

A modern concept in drug development is the use of pharmacophore hybrids to create compounds that have superior affinity and efficacy [50]. In considering the aforementioned facts, a reasonable and promising area of research in current medicinal chemistry is the design of novel small molecules with drug-like properties based on the pharmacologically attractive scaffolds of pyrazole and thiazolidinones [50]. Some recent synthetic studies of pyrazol–thiazolidines and related hybrids, along with their biological investigations, have been patented as necroptosis inhibitors [51], VHR protein tyrosine phosphatase inhibitors [52], Pin1-modulating compounds [53], and RNA-binding protein modulators [54]. The pyrazole–thiazolidinone hybrids were investigated for their ability to inhibit the TNF–a–TNFRc1 interaction [55], histone acetyltransferases inhibitors [56], COX inhibitors [57], and ADAMTS-5 enzymes [58]. 

As a result, in accordance with previously reported alkaline phosphatase inhibitors (Figure 1), we combined two scaffolds (pyrazole and thiazolidinone) to create a novel scaffold that was used in the ALP inhibition investigation. Moreover, to the best of our knowledge, pyrazolo-oxothiazolidine derivatives have never been researched as alkaline phosphatase inhibitors. Here, we described the design and synthesis of a novel class of ALP inhibitory agents which were also evaluated for antioxidant activity, cytotoxicity, and computational studies.

## 2. Results and Discussion

### 2.1. Chemistry

**Scheme 1:**
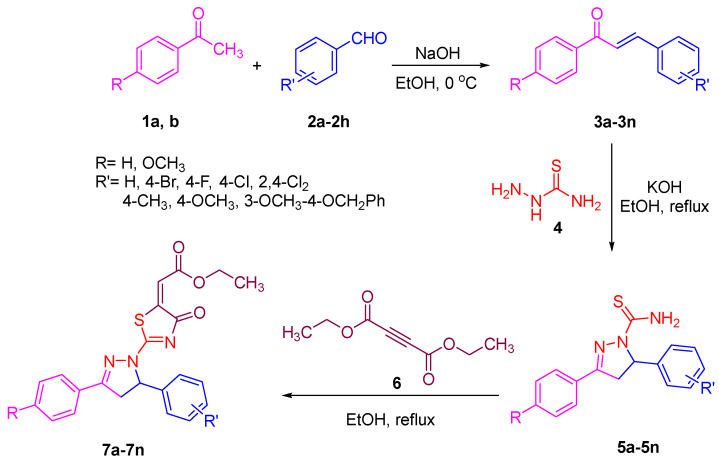
General synthesis of novel pyrazolo-oxothiazolidine derivatives (**7a**–**7n**).

**Figure 2:**
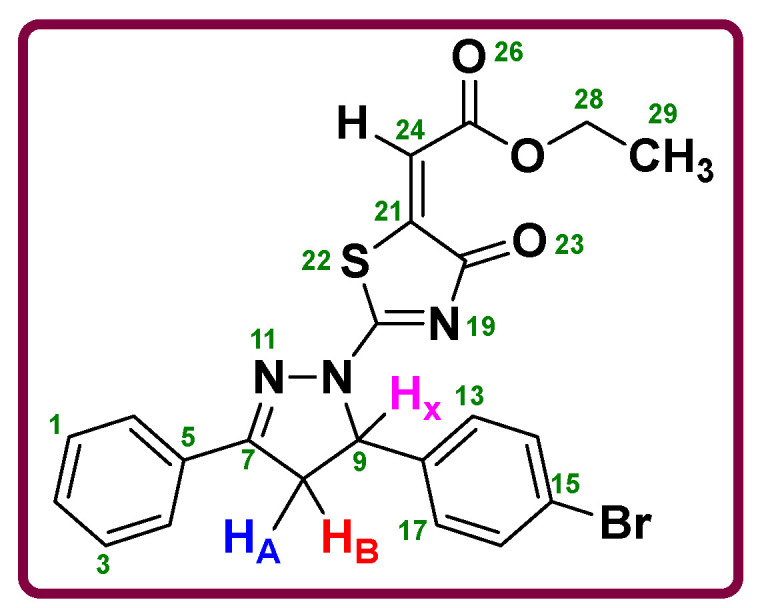
ABX system of the pyrazoline ring.

According to Scheme 1, a new series of pyrazolo-oxothiazolidine derivatives was synthesized in three steps. Initially, the Claisen–Schmidt condensation of acetophenone or *para*-methoxy acetophenone (**1a**, **b**) and aromatic aldehydes (**2a**–**2h**) in the presence of sodium hydroxide in ethanol resulted in 1,3-diphenylprop-2-en-1-ones (chalcones, **3a**–**3n**). Then, the intermolecular cyclization of different chalcone derivatives (**3a**–**3n**) with thiosemicarbazide (**4**) produced pyrazole compounds (**5a**–**5n**) in refluxing ethanol under basic conditions. Finally, the reaction of compounds **5a**–**5n** and diethyl acetylenedicarboxylate (**6**) is accomplished under reflux conditions in ethanol, to form pyrazolo-oxothiazolidine derivatives (**7a**–**7n**) in reasonable yields (80–92%). The structural confirmation of all synthesized compounds (**7a**–**7n**) was verified through the spectroscopic analysis utilizing FT-IR, ^1^H NMR, and ^13^C NMR. To comprehend the structural identification of the synthesized target compounds, we have presented a structural interpretation of one of the target compounds (**7a**). Molecule **7a** was obtained in 84% yield as a white solid with a melting point of 242–244 °C. FT-IR spectroscopy was used to examine the molecular vibrational studies to confirm various functionalities. The aliphatic C-H stretching vibrations were observed at 2974 cm^−1^. The stretching bands for conjugated C=O group appeared at 1694 cm^−1^. The other significant absorption bands in the IR spectrum emerged at 1602 (C=C, alkene), 1548, 1498 (C=C, aromatic), 1489 (C=N, stretching), 1218 (C-O, stretching), 1177, 1114, 1073, 1010 (C-N stretching and aromatic C-H out of plane bending), 823, 767, 757 (aromatic C-H out of plane bending), 689 (C-Br stretching), which prove the existence of functional groups such as pyrazole, ester, alkene, and aromatic in this molecule. The number of proton-containing atoms in the produced molecules was measured using ^1^H NMR spectroscopic techniques. There are two phenyl rings in the target compound **7a**. In the aromatic region, the two multiplet peaks at δ 7.14–7.18 (m, 2H, H-13 & 17), and 7.83–7.85 (m, 2H, H-14 & 16) belonged to the aromatic ring which holds the bromo group at the *para* position, whereas five protons of second phenyl ring represented by multiplet at δ 7.46–7.58 (m, 5H, H-1, 2, 3, 4 & 6). In the up-field region of the spectrum, the signals at δ 1.37 (t, *J* = 8 Hz, 3H, CH_3_) and 4.34 (q, *J* = 7.2 Hz, 2H, CH_2_), exposed the presence of methyl and methylene groups, which split triplet and quartet, respectively. At δ 6.93 (s, 1H, H-24), there was a distinct singlet signal for vinylic hydrogen. The typical ABX system, which is associated with the CH_2_ and CH protons of the pyrazoline ring, was clearly detected in the predicted regions. The H_A_ proton was observed at δ 3.43 (dd, *J* = 18, 4 Hz, 1H, H-8_A_), the H_B_ proton appeared at δ 4.03 (dd, *J* = 18, 12 Hz, 1H, H-8_B_), and the H_X_ emerged at 5.85 (dd, *J* = 11.2, 4 Hz, 1H, H-9), due to vicinal coupling with two magnetically non-equivalent protons of the methylene group at position 4 of the pyrazoline ring. Furthermore, the carbon backbone of the produced target compound **7a** was studied using the ^13^C NMR technique. Due to some duplets for magnetically equivalent carbons that reduced the total number of resonances in the molecule, the ^13^C NMR spectra showed a total of eighteen carbon resonances. Two resonances for the phenyl ring which holds the bromo group at the para position were observed at δ 127.68 (C-13 & C-17) and 132.39 (C-14 & C-16), while another phenyl ring emerged at δ 127.52 (C-4 & C-6), 129.11 (C-1 & C-3), and 129.43 (C-2). The two carbonyl carbons (C-25) and (C-20) appeared at δ 166.59 and 173.81 ppm, whereas two imine carbons (C-7) and (C-18) were observed at δ 160.79 and 178.68 ppm. Four quaternary signals were characterized at δ 122.65 (C-15), 132.16 (C-5), 138.15 (C-12), and 146.52 (C-21). One signal at δ 118.17 ppm belonged to the methine carbon (C-24). There were four peaks in the aliphatic region that were distinguished by the methyl and methylene carbons at positions 14.23 (C-29), 43.30 (C-8), 61.75 (C-28), and 63.38. (C-9) (Figure 2).

### 2.2. Biological Evaluations

#### 2.2.1. Alkaline Phosphatase Activity and Structural Activity Relationship (SAR)

The synthesized chemical compounds (**7a**–**7n**) were evaluated against the calf intestinal alkaline phosphatase (CIAP) enzyme inhibition for the IC_50_ evaluation. Table 1 gives an overview of the CIAP results. Monopotassium phosphate (KH_2_PO_4_) was employed in this experiment as a positive control to evaluate the performance of our synthetic compounds. Interestingly, all these compounds showed extremely strong inhibitory activity against this enzyme, as evidenced by their lower IC_50_ (0.045–2.987 μM) values in comparison to the standard reference KH_2_PO_4_, which had an IC_50_ value of 5.242 ± 0.472 μM. Though the observed activity is the resultant of the entire molecule, the effect of various aryl groups on the inhibitory potential allowed for the recognition of a limited structure-activity relationship (SAR). To make the classification of the structure activity relationship (SAR) more understandable, we divide it into three categories. The first five compounds **7**(**a**–**d**), and **7l**, while having the unsubstituted phenyl ring scaffold, differ in their second phenyl ring derivatives. It observed that compounds **7a** and **7l**, which have halogen groups (Br and Cl) at the *para* position, showed better activity when compared to other derivatives (**7b**, **7c**, **7d**), which have electron-donating groups at the *para* position. However, a reverse trend was noticed in the second set of seven compounds **7e**, **7f**, **7g**, **7h**, **7i**, **7k**, and **7m**, which all possess a *para*-methoxy phenyl ring, and the second phenyl ring has various derivatives. Among halogenated molecules (**7h**, **7m**, **7f**, and **7i**), compound **7h** with bromo group at *para* position exhibited less activity probably because a bulky bromo group decreases the inhibitory activity. Compound **7f** with chloro group at *ortho* and *para* position showed lower inhibitory potential as compared to **7m** in which chloro group was at *para* position, and presumably due to steric crowding prevents the good interaction with the active site of the enzyme. Compounds **7i** and **7m**, which have fluoro and chloro groups in the *para* positions, respectively, displayed a similar inhibitory effect. Additionally, the methyl, methoxy-benzyloxy, and methoxy groups found in compounds **7e**, **7g**, and **7k** in this category showed overall the strongest inhibitory potentials against the target enzyme. So, it can be concluded that, when one phenyl ring has a *para*-methoxy group, the electron-withdrawing groups significantly disrupted the electronic density of the aromatic ring, and more effective interactions are made by electron-donating substances with the enzyme. Surprisingly, the most effective molecule **7g** (IC_50_ = 0.045 ± 0.004 μM) comprised both benzyloxy and methoxy groups, which showed synergistic effects. This might be caused by the ability of both groups to donate electrons via resonance and created more hydrogen bonds. Finally, between compounds **7n** and **7j**, compound **7j** (IC_50_ = 0.434 ± 0.011 μM) with only one methoxy group at *para* position showed almost six-fold better inhibitory potential compared to **7n** (IC_50_ = 2.987 ± 0.734 μM) having unsubstituted phenyl rings. It means that compared to an unsubstituted compound, the presence of an electron-donating substituent on the phenyl group makes the molecule a more suitable inhibitor. Overall, all of the derivatives have noticeable inhibitory activity, particularly compound **7g** possessing the strongest effect compared to reference standard. As a consequence of these findings, it can be concluded that phenyl rings need to contain electron-rich species like methoxy and benzyloxy groups in order to have alkaline phosphatase inhibitory activity. Therefore, compound **7g** could be a perfect choice for medicinal purposes.

#### 2.2.2. Kinetic Mechanism for CIAP

**Figure 3:**
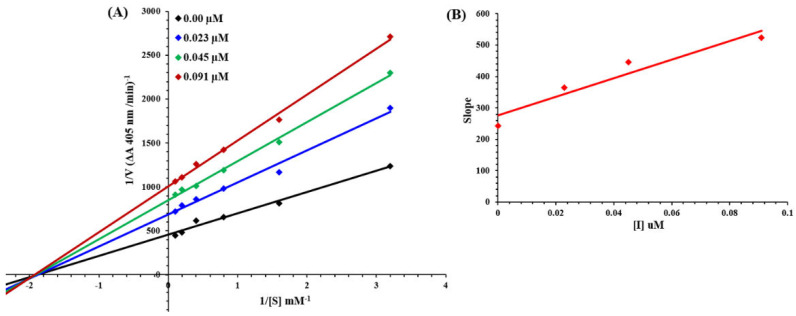
Lineweaver-Burk plots for the inhibition of alkaline phosphatase in the presence of compound **7g** (**A**). The insets represent the plot of the slope as opposed to inhibitor concentrations to determine the inhibition constant (**B**). The lines were drawn using linear least squares fit.

Presently, the most active compound **7g** was considered for the mode of inhibition of CIAP. The capacity of the compound to inhibit the free enzyme and enzyme-substrate complex was determined in terms of EI and ESI constants, respectively. The kinetic research of the enzyme by the Lineweaver–Burk plot of 1/V versus substrate p-NPP 1/[S] in the presence of different inhibitor concentrations gave a series of straight lines as shown in Figure 3A. The results of compound **7g** showed that the compound intersected within the second quadrant. The analysis confirmed that V_max_ was reduced to new growing doses of inhibitor, on the other hand K_m_ stays the same. This behavior suggests that compound **7g** inhibits the alkaline phosphatase non-competitively to shape an enzyme-inhibitor complex. A secondary plot of slope against the concentrations of inhibitor confirmed enzyme-inhibitor dissociation constant (*K*i) Figure 3B. The kinetic results are presented in Table 2.

#### 2.2.3. Free Radical Scavenging Activity

**Figure 4:**
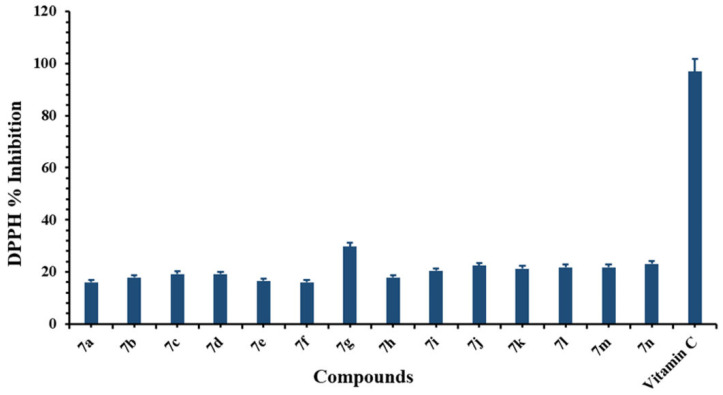
Free radical % scavenging activity of synthesized compounds (**7a**–**7n**). Values were depicted as mean ± SEM.

All synthesized target compounds were assessed for 2, 2-diphenyl-1-picrylhydrazyl (DPPH) free radical scavenging ability. From our results, all of the compounds showed activity (Figure 4), but the most effective compound (**7g**) showed a bit of inhibition (29.7%) compared to the others at the concentration of 100 µg/mL. Ascorbic acid (vitamin C) was employed as a reference to evaluate the radical scavenging activity.

#### 2.2.4. Cell Viability

**Figure 5:**
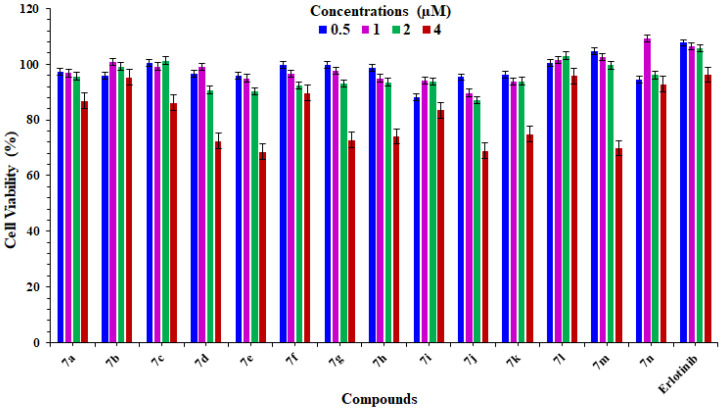
Cell viability results of synthesized compounds (**7a**–**7n**) on the human osteosarcoma MG-63 cell lines using the MTT assay at different concentrations for 24 h.

In this experiment, all fourteen pyrazolo-oxothiazolidine derivatives (**7a**–**7n**) after confirmation activity against in vitro ALP enzyme inhibition were carried out to check their toxic effect on the human osteosarcoma MG-63 cell lines using the MTT assay technique. At first, four concentrations (0.5, 1, 2, and 4 µM) were chosen for each compound, along with a positive control (erlotinib), and all compounds and the standard drug were treated and incubated for 24 h. Normal control was considered to be 100% viable without any treatment. The viability of the cells was examined after incubation, and the results are presented in (Figure 5). From these results, it is clear that all compounds have no toxic effect on the cells, and even at the concentration of 4 µM, the cells were viable up to >70% except the compounds **7e**, **7j**, and **7m**. However, at the first three treatments, all the compounds along with the standard drug showed >90% viability. These findings suggest that these compounds could be employed as a safe target for drug design within pharmaceutical approaches.

### 2.3. In Silico

#### 2.3.1. Biochemical Properties and Lipinski’s Rule of Five (RO5) Validation

The biochemical properties of all synthesized compounds (**7a**–**7n**) were predicted and analyzed through computational resources. The synthesized target compounds (**7a**–**7n**) were validated through RO5 analysis. The standard parameters in RO5 showed that the molecular mass and log*P* values must be less than 500 (g/mol) and 5, respectively. Moreover, the compounds should possess no greater than 10 HBA and 5 HBD, correspondingly. According to the literature, excessive values of HBA and HBD result in poor permeation [59,60]. The hydrogen-bonding capacity has been considered a significant parameter for drug permeability. Our results demonstrated the synthesized ligands possessed <10 HBA and <5 HBD values, which were comparable to standard values. However, the log*P* values in most compounds were also comparable with standard values (>5). The molecular weight (g/mol) of all compounds exceeded the standard value (Table 3). However, there are many examples available which deviate RO5 violation amongst the existing drugs [61,62]. Polar surface area (PSA) is also considered a good descriptor for characterizing drug absorption, including intestinal absorption, bioavailability, and blood–brain barrier penetration. Our predicted results showed that all compounds possess <140 Å^2^ values, which showed their good lead-like behavior. The drug-likeness score is a mixture of a complex balance of various molecular properties and structural features of chemical compounds, which determine whether the molecule is like as-known drugs [63]. Our results showed that all the synthetic compounds (**7a**–**7n**) showed good drug score values. The majority of the compounds exhibited positive drug score values, whereas **7b** and **7n** possessed negative score values (Table 3).

#### 2.3.2. Structural Analysis and Physiochemical Properties of ALP

**Figure 6:**
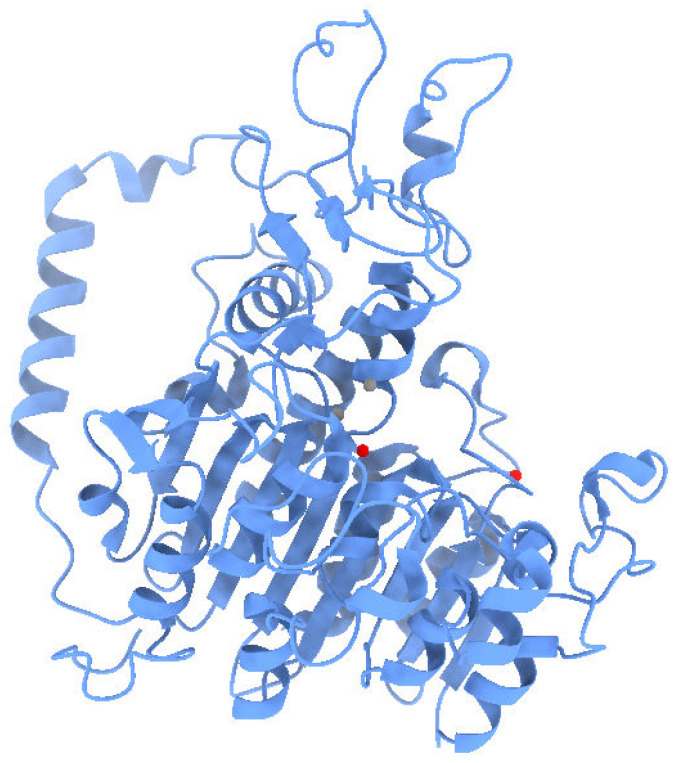
Three-dimensional structure of ALP.

Human placental alkaline phosphatase is a class of hydrolase single-chain protein, having 539 amino acids with embedded zinc and magnesium ions at different structural sites. The VADAR analysis showed the overall protein architecture consists of 31% helices, 26% β sheets, and 42% coils. Moreover, the Ramachandran plot indicated that 96.4% of residues were present in favored regions which showed the precision of phi (ϕ) and psi (ψ) angles among the coordinates of ALP (Figure 6).

#### 2.3.3. Binding Energy Evaluation of Compounds

To predict the best conformational position within the active region of the target protein, all the synthesized ligands (**7a**–**7n**) were docked against ALP. All the generated docked complexes were analyzed based on minimum energy values (kcal/mol) and hydrogen/hydrophobic interactions. Docking results justified that all the synthesized target compounds exhibited good docking binding affinities with ALP. Both **7d** (−8.3 Kcal/mol) and **7g** (−8.1 Kcal/mol) exhibited higher binding energy values as compared to other synthesized ligands. The remaining compounds showed different binding energy values, and most of the ligands were bound within the active region of the target protein. The basic chemical nuclei of all the synthesized compounds were the same; therefore, most ligands showed a good binding affinity with the target protein (Table 4).

#### 2.3.4. Binding Interaction of **7g** against ALP

**Figure 7:**
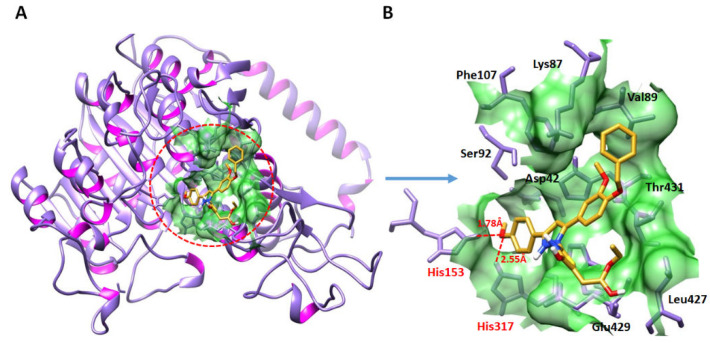
Docking complex of **7g** against ALP (**A**,**B**).

Based on in vitro (IC_50_) results, the **7g**-docked complex was selected to understand the binding interaction behavior of the compound against ALP. Docking analysis showed that **7g** was actively confined within the active binding region of ALP (Figure 7). In detail, the SAR study showed that four hydrogen bonds were observed in the **7g**-docking complex. The oxygen atom of **7g** forms two hydrogen bonds with His_153 and His_317 having bond lengths 1.78 and 2.55 Å, respectively. The importance of these amino acid residues in binding with other ALP inhibitors has also been well documented [64,65]. The comparative binding energy and SAR analysis showed a significance of **7g** and may be considered as a potent inhibitor of the enzyme. The rest of all docking complexes have been shown in Supplementary Data (Figures S43–S56).

## 3. Methods and Materials

### 3.1. Chemicals and Instruments

The MPA 160 apparatus of Fisher Scientific [Waltham, MA, USA] was used to determine the melting points (uncorrected) of the synthesized compounds. The FT-IR spectra was obtained using a Frontier IR spectrophotometer (Perkin Elmer, Waltham, MA, USA). ^1^H NMR and ^13^C NMR spectra of the compounds were recorded on a Bruker Avance III (Karlsruhe, Germany) at 400 and 100 MHz spectrophotometers using CDCl_3_ as solvent. Chemical shifts were reported in parts per million (ppm) (δ). A thin layer chromatography (TLC) method was used to monitor the progress of the reaction. All commercially available chemicals and reagents were utilized without further purification.

### 3.2. General Procedure for the Synthesis of the Compounds

#### 3.2.1. Procedure for Synthesis of Chalcones (**3a**–**3n**)

Acetophenone or 4-methoxyacetophenone (**1a** and **1b**, 1 mmol) was dissolved in approximately 10 mL of absolute ethyl alcohol and stirred for 5 min in an ice bath, as shown in Scheme 1. The sodium hydroxide (1.1 mmol) was then added portion-wise to the reaction flask. Following that, various substituted aromatic aldehydes (**2a**–**2h**, 1 mmol) were slowly added to the reaction mixture and stirred for 24 h in an ice bath. When the reaction was completed (determined by thin layer chromatography) the reaction mixture was poured into crushed ice and neutralized with hydrochloric acid. The obtained precipitate was stirred for 30 min at room temperature and then filtered, washed with cold water/ethanol, and dried to afford the desired chalcones (**3a**–**3n**) [66].

#### 3.2.2. Procedure for Synthesis of 1-Thiocarbamoyl Pyrazole Derivatives (**5a**–**5n**)

As displayed in Scheme 1, 1.5 mmol thiosemicarbazide (**4**) and absolute ethanol were mixed for 10 min. Then, under the nitrogen atmosphere, 1.1 mmol potassium hydroxide was added and stirred for another 5–10 min, followed by the addition of corresponding chalcones (**3a**-**3n**, 1 mmol). The reaction was subsequently continued under reflux for 8 h until the reaction was completed (monitored by thin layer chromatography). The product was poured into crushed ice, neutralized with hydrochloric acid, stirred for 30 min, then filtered, washed with cold ethanol, and dried to obtain the 1-thiocarbamoyl pyrazole derivatives (**5a**–**5n**) [67].

#### 3.2.3. Procedure for Synthesis of 1-Thiazolyl-2-Pyrazoline Derivatives (**7a**–**7n**)

As presented in Scheme 1, to a suspension of 1-thiocarbamoyl pyrazole compounds (**5a**–**5n**, 1mmol) in absolute ethanol, diethyl acetylenedicarboxylate (**6**, 1mmol) was added and followed by refluxing for 4 h. The reaction mixture was cooled at room temperature after completion (as confirmed by thin layer chromatography), and the precipitate was filtered and recrystallized from ethanol to achieve the desired target compounds (**7a**–**7n**) in good yields (80–92%).

##### Ethyl-2-(2-(5-(4-bromophenyl)-3-phenyl-4,5-dihydro-1H-pyrazol-1-yl)-4-oxothiazol-5(4H)-ylidene)acetate (**7a**)


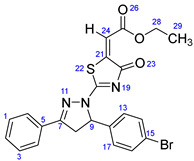


Yellow solid, isolated yield: 84%; mp: 242–244 °C. Figure S1: IR (KBr) ν (cm^−1^): 2974 (C-H, alkane, stretching), 1694 (C=O, ester), 1602 (C=C, alkene), 1548, 1498 (C=C, aromatic), 1489 (C=N, stretching), 1218 (C-O, stretching), 1177, 1114, 1073, 1010 (C-N stretching and aromatic C-H out of plane bending), 823, 767, 757 (aromatic C-H out of plane bending), 689 (C-Br stretching). Figure S2: ^1^H NMR (400 MHz, CDCl_3_) δ (ppm): 1.37 (t, *J =* 8 Hz, 3H, CH_3_-29), 3.43 (dd, *J =* 18, 4 Hz, 1H, H-8_A_), 4.03 (dd, *J =* 18, 12 Hz, 1H, H-8_B_), 4.34 (q, *J =* 7.2 Hz, 2H, CH_2_-28), 5.85 (dd, *J =* 11.2, 4 Hz, 1H, H-9), 6.93 (s, 1H, H-24), 7.14–7.18 (m, 2H, H-13 & 17), 7.46–7.58 (m, 5H, H-1, 2, 3, 4 & 6), 7.83–7.85 (m, 2H, H-14 & 16). Figure S3: ^13^C NMR (100 MHz, CDCl_3_) δ (ppm): 14.23 (C-29), 43.30 (C-8), 61.75 (C-28), 63.38 (C-9), 118.17 (C-24), 122.65 (C-15), 127.52 (C-4 & 6), 127.68 (C-13 & 17), 129.11 (C-1 & 3), 129.43 (C-2), 132.16 (C-5), 132.39 (C-14 & 16), 138.15 (C-12), 146.52 (C-21), 160.79 (C-7), 166.59 (C-25), 173.81 (C-20), 178.68 (C-18).

##### Ethyl-2-(4-oxo-2-(3-phenyl-5-(p-tolyl)-4,5-dihydro-1H-pyrazol-1-yl)thiazol-5(4H)-ylidene)acetate (**7b**)


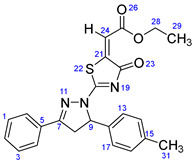


Yellow solid, isolated yield: 81%; mp: 226–228 °C. Figure S4: IR (KBr) ν (cm^−1^): 2979 (C-H, alkane, stretching), 1694 (C=O, ester), 1602 (C=C, alkene), 1554, 1495 (C=C, aromatic), 1447 (C=N, stretching), 1225 (C-O, stretching), 1184, 1127, 1025 (C-N stretching and aromatic C-H out of plane bending), 833, 769, 757 (aromatic C-H out of plane bending). Figure S5: ^1^H NMR (400 MHz, CDCl_3_) δ (ppm): 1.37 (t, *J =* 8 Hz, 3H, CH_3_-29), 2.33 (s, 3H, CH_3_-31), 3.45 (dd, *J =* 16, 4 Hz, 1H, H-8_A_), 4.00 (dd, *J =* 18, 12 Hz, 1H, H-8_B_), 4.33 (q, *J =* 8 Hz, 2H, CH_2_-28), 5.86 (dd, *J =* 12, 4 Hz, 1H, H-9), 6.92 (s, 1H, H-24), 7.14–7.19 (m, 4H, H-13, 14, 16 & 17), 7.48–7.58 (m, 3H, H-1, 2 & 3), 7.85–7.87 (m, 2H, H-4 & 6). Figure S6: ^13^C NMR (100 MHz, CDCl_3_) δ (ppm): 14.24 (C-29), 21.16 (C-31), 43.46 (C-8), 61.67 (C-28), 63.85 (C-9), 117.79 (C-24), 125.86 (C-4 & 6), 127.51 (C-13 & 17), 129.05 (C-1 & 3), 129.68 (C-2), 129.85 (C-14 & 16), 131.99 (C-5), 136.25 (C-12), 138.48 (C-15), 146.81 (C-21), 161.02 (C-7), 166.66 (C-25), 173.59 (C-20), 178.81 (C-18).

##### Ethyl-2-(2-(5-(4-methoxyphenyl)-3-phenyl-4,5-dihydro-1H-pyrazol-1-yl)-4-oxothiazol-5(4H)-ylidene)acetate (**7c**)


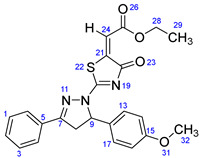


Yellow solid, isolated yield: 82%; mp: 236–238 °C. Figure S7: IR (KBr) ν (cm^−1^): 2937 (C-H, alkane, stretching), 1693 (C=O, ester), 1601 (C=C, alkene), 1539, 1515 (C=C, aromatic), 1455 (C=N, stretching), 1243 (C-O, stretching), 1179, 1165, 1095, 1022 (C-N stretching and aromatic C-H out of plane bending), 827, 767, 720 (aromatic C-H out of plane bending). Figure S8: ^1^H NMR (400 MHz, CDCl_3_) δ (ppm): 1.37 (t, *J =* 8 Hz, 3H, CH_3_-29), 3.47 (dd, *J =* 18, 4 Hz, 1H, H-8_A_), 3.80 (s, 3H, OCH_3_-32), 3.99 (dd, *J =* 18, 12 Hz, 1H, H-8_B_), 4.33 (q, *J =* 8 Hz, 2H, CH_2_-28), 5.84 (dd, *J =* 10, 4 Hz, 1H, H-9), 6.85–6.89 (m, 2H, H-14 & 16), 6.92 (s, 1H, H-24), 7.21–7.25 (m, 2H, H-13 & 17), 7.49–7.58 (m, 3H, H-1, 2 & 3), 7.85–7.88 (m, 2H, H-4 & 6). Figure S9: ^13^C NMR (100 MHz, CDCl_3_) δ (ppm): 14.24 (C-29), 43.32 (C-8), 55.33 (C-32), 61.67 (C-28), 63.62 (C-9), 114.50 (C-14 & 16), 117.78 (C-24), 127.44 (C-4 & 6), 127.51 (C-13 & 17), 129.07 (C-1 & 3), 129.69 (C-2), 131.25 (C-12), 132.00 (C-5), 146.79 (C-21), 159.72 (C-7), 161.05 (C-15), 166.66 (C-25), 173.55 (C-20), 178.84 (C-18).

##### Ethyl-2-(2-(5-(4-(benzyloxy)-3-methoxyphenyl)-3-phenyl-4,5-dihydro-1H-pyrazol-1-yl)-4-oxothiazol-5(4H)-ylidene)acetate (**7d**)


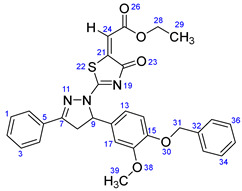


Yellow solid, isolated yield: 84%; mp: 202–204 °C. Figure S10: IR (KBr) ν (cm^−1^): 2951 (C-H, alkane, stretching), 1703, 1686 (C=O, stretching), 1600 (C=C, alkene), 1540, 1497 (C=C, aromatic), 1448 (C=N, stretching), 1230 (C-O, stretching), 1189, 1166, 1134, 1115, 1024 (C-N stretching and aromatic C-H out of plane bending), 863, 762, 737 (aromatic C-H out of plane bending). Figure S11: ^1^H NMR (400 MHz, CDCl_3_) δ (ppm): 1.37 (t, *J =* 8 Hz, 3H, CH_3_-29), 3.46 (dd, *J =* 16, 4 Hz, 1H, H-8_A_), 3.87 (s, 3H, OCH_3_-39), 3.97 (dd, *J =* 16, 12 Hz, 1H, H-8_B_), 4.34 (q, *J =* 8 Hz, 2H, CH_2_-28), 5.13 (s, 2H, CH_2_-31), 5.84 (dd, *J =* 10, 4 Hz, 1H, H-9), 6.73 (dd, *J =* 8.4, 2 Hz, 1H, H-14), 6.81 (s, 1H, H-17), 6.83–6.84 (m, 1H, H-13), 6.93 (s, 1H, H-24), 7.30–7.43 (m, 5H, H-1, 3, 34, 35 & 36), 7.49–7.58 (m, 3H, H-2, 33 & 37), 7.85 (d, *J =* 8 Hz, 2H, H-4 & 6). Figure S12: ^13^C NMR (100 MHz, CDCl_3_) δ (ppm): 14.24 (C-29), 43.36 (C-8), 56.22 (C-39), 61.70 (C-28), 63.82 (C-9), 71.04 (C-31), 109.93 (C-17), 114.18 (C-14), 117.88 (C-24), 117.92 (C-13), 127.23 (C-4 & 6), 127.51 (C-33 & 37), 127.91 (C-35), 128.60 (C-34 & 36), 129.07 (C-1 & 3), 129.64 (C-2), 132.03 (C-12), 132.12 (C-5), 136.91 (C-32), 146.74 (C-21), 148.42 (C-15), 150.01 (C-16), 161.08 (C-7), 166.66 (C-25), 173.65 (C-20), 178.79 (C-18).

##### Ethyl-2-(2-(3-(4-methoxyphenyl)-5-(p-tolyl)-4,5-dihydro-1H-pyrazol-1-yl)-4-oxothiazol-5(4H)-ylidene)acetate (**7e**)


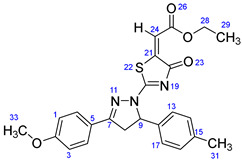


Yellow solid, isolated yield: 86%; mp: 208–210 °C. Figure S13: IR (KBr) ν (cm^−1^): 2967 (C-H, alkane, stretching), 1687 (C=O, ester), 1607 (C=C, alkene), 1557, 1512 (C=C, aromatic), 1463 (C=N, stretching), 1250, 1226 (C-O, stretching), 1172, 1113, 1027 (C-N stretching and aromatic C-H out of plane bending), 830, 769, 720 (aromatic C-H out of plane bending). Figure S14: ^1^H NMR (400 MHz, CDCl_3_) δ (ppm): 1.37 (t, *J =* 8 Hz, 3H, CH_3_-29), 2.33 (s, 3H, CH_3_-31), 3.41 (dd, *J =* 20, 4 Hz, 1H, H-8_A_), 3.91 (s, 3H, OCH_3_-33), 3.96 (dd, *J =* 16, 12 Hz, 1H, H-8_B_), 4.33 (q, *J =* 8 Hz, 2H, CH_2_-28), 5.83 (dd, *J =* 12, 4 Hz, 1H, H-9), 6.91 (s, 1H, H-24), 6.98–7.02 (m, 2H, H-1 & 3), 7.14–7.18 (m, 4H, H-13, 14, 16 & 17), 7.78–7.82 (m, 2H, H-4 & 6). Figure S15: ^13^C NMR (100 MHz, CDCl_3_) δ (ppm): 14.23 (C-29), 21.15 (C-31), 43.46 (C-8), 55.55 (C-33), 61.62 (C-28), 63.76 (C-9), 114.47 (C-1 & 3), 117.52 (C-24), 122.23 (C-5), 125.86 (C-4 & 6), 129.34 (C-13 & 17), 129.81 (C-14 & 16), 136.34 (C-12), 138.41 (C-15), 147.01 (C-21), 160.74 (C-7), 162.68 (C-2), 166.72 (C-25), 172.97 (C-20), 178.77 (C-18).

##### Ethyl-2-(2-(5-(2,4-dichlorophenyl)-3-(4-methoxyphenyl)-4,5-dihydro-1H-pyrazol-1-yl)-4-oxothiazol-5(4H)-ylidene)acetate (**7f**)


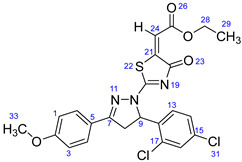


White solid, isolated yield: 90%; mp>260 °C. Figure S16: IR (KBr) ν (cm^−1^): 2974 (C-H, alkane, stretching), 1688 (C=O, ester), 1607 (C=C, alkene), 1591, 1547 (C=C, aromatic), 1513 (C=N, stretching), 1254, 1227 (C-O, stretching), 1177, 1119, 1099, 1028 (C-N stretching and aromatic C-H out of plane bending), 856 (C-Cl, stretching), 837, 770, 730 (aromatic C-H out of plane bending). Figure S17: ^1^H NMR (400 MHz, CDCl_3_) δ (ppm): 1.38 (t, *J =* 8 Hz, 3H, CH_3_-29), 3.29 (dd, *J =* 18, 4 Hz, 1H, H-8_A_), 3.90 (s, 3H, OCH_3_-32), 4.07 (dd, *J =* 20, 12 Hz, 1H, H-8_B_), 4.35 (q, *J =* 8 Hz, 2H, CH_2_-28), 6.11 (dd, *J =* 12, 4 Hz, 1H, H-9), 6.95–6.99 (m, 4H, H-1, 3, 13 & 24), 7.21 (dd, *J =* 8.4, 1 Hz, 1H, H-14), 7.47 (d, *J =* 2 Hz, 1H, H-16), 7.75–7.79 (m, 2H, H-4 & 6). Figure S18: ^13^C NMR (100 MHz, CDCl_3_) δ (ppm): 14.24 (C-29), 42.61 (C-8), 55.56 (C-33), 61.47 (C-28), 61.77 (C-9), 114.49 (C-1 & 3), 118.23 (C-24), 121.85 (C-13), 127.03 (C-14), 127.82 (C-5), 129.35 (C-4 & 6), 130.22 (C-17), 132.47 (C-15), 134.82 (C-12), 134.87 (C-16), 146.63 (C-21), 160.61 (C-7), 162.82 (C-2), 166.64 (C-25), 173.49 (C-20), 178.64 (C-18).

##### Ethyl-2-(2-(5-(4-(benzyloxy)-3-methoxyphenyl)-3-(4-methoxyphenyl)-4,5-dihydro-1H-pyrazol-1-yl)-4-oxothiazol-5(4H)-ylidene)acetate (**7g**)


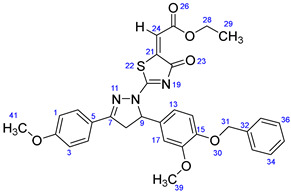


Yellow solid, isolated yield: 85%; mp: 228–230 °C. Figure S19: IR (KBr) ν (cm^−1^): 2996, 2948 (C-H, alkane, stretching), 1703, 1684 (C=O, stretching), 1610 (C=C, alkene), 1597, 1549 (C=C, aromatic), 1514 (C=N, stretching), 1262, 1228 (C-O, stretching), 1192, 1174, 1135, 1117, 1016 (C-N stretching and aromatic C-H out of plane bending), 864, 834, 746, 724 (aromatic C-H out of plane bending). Figure S20: ^1^H NMR (400 MHz, CDCl_3_) δ (ppm): ^1^H NMR (400 MHz, CDCl_3_) δ (ppm): 1.37 (t, *J =* 8 Hz, 3H, CH_3_-29), 3.41 (dd, *J =* 18, 4 Hz, 1H, H-8_A_), 3.87 (s, 3H, OCH_3_-39), 3.90 (s, 3H, OCH_3_-41), 3.94 (dd, *J =* 18, 12 Hz, 1H, H-8_B_), 4.33 (q, *J =* 8 Hz, 2H, CH_2_-28), 5.13 (s, 2H, CH_2_-31), 5.81 (dd, *J =* 12, 4 Hz, 1H, H-9), 6.73 (dd, *J =* 8, 4 Hz, 1H, H-14), 6.80 (s, 1H, H-17), 6.83–6.84 (m, 1H, H-13), 6.92 (s, 1H, H-24), 6.98–7.01 (m, 2H, H-1 & 3), 7.30–7.43 (m, 5H, H-33, 34, 35, 36 & 37), 7.77–7.81 (m, 2H, H-4 & 6). Figure S21: ^13^C NMR (100 MHz, CDCl_3_) δ (ppm): 14.24 (C-29), 43.35 (C-8), 55.55 (C-41), 56.21 (C-39), 61.64 (C-28), 63.73 (C-9), 71.05 (C-31), 109.95 (C-17), 114.17 (C-14), 114.48 (C-1 & 3), 117.59 (C-24), 117.94 (C-13), 122.18 (C-5), 127.23 (C-4 & 6), 127.89 (C-35), 128.59 (C-33 & 37), 129.34 (C-34 & 36), 132.24 (C-12), 136.94 (C-32), 146.95 (C-21), 148.38 (C-15), 149.99 (C-16), 160.81 (C-7), 162.71 (C-2), 166.71 (C-25), 173.01 (C-20), 178.76 (C-18).

##### Ethyl-2-(2-(5-(4-bromophenyl)-3-(4-methoxyphenyl)-4,5-dihydro-1H-pyrazol-1-yl)-4-oxothiazol-5(4H)-ylidene)acetate (**7h**)


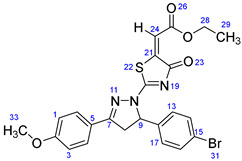


Yellow solid, isolated yield: 89%; mp: 218–220 °C. Figure S22: IR (KBr) ν (cm^−1^): 2969 (C-H, alkane, stretching), 1687 (C=O, ester), 1607 (C=C, alkene), 1588, 1542 (C=C, aromatic), 1511, 1488 (C=N, stretching), 1308, 1249 (C-O, stretching), 1177, 1105, 1030, 1008 (C-N stretching and aromatic C-H out of plane bending), 844, 836, 814, 768, 724 (aromatic C-H out of plane bending), 658 (C-Br stretching). Figure S23: ^1^H NMR (400 MHz, CDCl_3_) δ (ppm): 1.37 (t, *J =* 8 Hz, 3H, CH_3_-29), 3.39 (dd, *J =* 16, 4 Hz, 1H, H-8_A_), 3.91 (s, 3H, OCH_3_-33), 3.99 (dd, *J =* 18, 12 Hz, 1H, H-8_B_), 4.34 (q, *J =* 8 Hz, 2H, CH_2_-28), 5.82 (dd, *J =* 10, 4 Hz, 1H, H-9), 6.92 (s, 1H, H-24), 6.99–7.02 (m, 2H, H-1 & 3), 7.15–7.18 (m, 2H, H-13 & 17), 7.46–7.50 (m, 2H, H-14 & 16), 7.77–7.80 (m, 2H, H-4 & 6). Figure S24: ^13^C NMR (100 MHz, CDCl_3_) δ (ppm): 14.23 (C-29), 43.29 (C-8), 55.57 (C-33), 61.70 (C-28), 63.29 (C-9), 114.53 (C-1 & 3), 117.89 (C-24), 121.96 (C-15), 122.59 (C-5), 127.69 (C-4 & 6), 129.37 (C-13 & 17), 132.36 (C-14 & 16), 138.25 (C-12), 146.72 (C-21), 160.48 (C-7), 162.81 (C-2), 166.65 (C-25), 173.20 (C-20), 178.66 (C-18).

##### Ethyl-2-(2-(5-(4-fluorophenyl)-3-(4-methoxyphenyl)-4,5-dihydro-1H-pyrazol-1-yl)-4-oxothiazol-5(4H)-ylidene)acetate (**7i**)


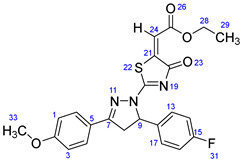


Yellow solid, isolated yield: 88%; mp: 210–212 °C. Figure S25: IR (KBr) ν (cm^−1^): 2975 (C-H, alkane, stretching), 1686 (C=O, ester), 1604 (C=C, alkene), 1544, 1510 (C=C, aromatic), 1474 (C=N, stretching), 1311, 1222 (C-O, stretching), 1184 (C-F, stretching), 1174, 1111, 1039 (C-N stretching and aromatic C-H out of plane bending), 858, 831, 809, 769, 729 (aromatic C-H out of plane bending). Figure S26: ^1^H NMR (400 MHz, CDCl_3_) δ (ppm): 1.37 (t, *J =* 8 Hz, 3H, CH_3_-29), 3.40 (dd, *J =* 16, 4 Hz, 1H, H-8_A_), 3.91 (s, 3H, OCH_3_-33), 3.98 (dd, *J =* 16, 12 Hz, 1H, H-8_B_), 4.33 (q, *J =* 8 Hz, 2H, CH_2_-28), 5.85 (dd, *J =* 12, 4 Hz, 1H, H-9), 6.92 (s, 1H, H-24), 6.99–7.06 (m, 4H, H-1, 3, 14 & 16), 7.25–7.27 (m, 2H, H-13 & 17), 7.78–7.82 (m, 2H, H-4 & 6). Figure S27: ^13^C NMR (100 MHz, CDCl_3_) δ (ppm): 14.23 (C-29), 43.39 (C-8), 55.57 (C-33), 61.68 (C-28), 63.21 (C-9), 114.52 (C-1 & 3), 116.16 (d, *J =* 21 Hz, C-14 & 16), 117.79 (C-24), 122.03 (C-5), 127.87 (d, *J =* 8.8 Hz, C-13 & 17), 129.36 (C-4 & 6), 135.09 (d, *J =* 3 Hz, C-12), 146.77 (C-21), 160.57 (C-7), 162.62 (d, *J =* 246 Hz, C-15), 162.78 (C-2), 166.66 (C-25), 173.14 (C-20), 178.71 (C-18).

##### Ethyl-2-(2-(3-(4-methoxyphenyl)-5-phenyl-4,5-dihydro-1H-pyrazol-1-yl)-4-oxothiazol-5(4H)-ylidene)acetate (**7j**)


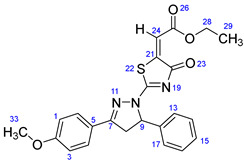


Yellow solid, isolated yield: 81%; mp: 167–169 °C. Figure S28: IR (KBr) ν (cm^−1^): 2976 (C-H, alkane, stretching), 1686 (C=O, ester), 1606 (C=C, alkene), 1592, 1552 (C=C, aromatic), 1511, 1464 (C=N, stretching), 1309, 1249 (C-O, stretching), 1176, 1114, 1026 (C-N stretching and aromatic C-H out of plane bending), 858, 834, 768, 722 (aromatic C-H out of plane bending). Figure S29: ^1^H NMR (400 MHz, CDCl_3_) δ (ppm): 1.37 (t, *J =* 8 Hz, 3H, CH_3_-29), 3.42 (dd, *J =* 20, 4 Hz, 1H, H-8_A_), 3.90 (s, 3H, OCH_3_-33), 3.98 (dd, *J =* 18, 12 Hz, 1H, H-8_B_), 4.33 (q, *J =* 8 Hz, 2H, CH_2_-28), 5.87 (dd, *J =* 12, 4 Hz, 1H, H-9), 6.91 (s, 1H, H-24), 6.98–7.01 (m, 2H, H-1 & 3), 7.26–7.28 (m, 2H, H-14 & 16), 7.31–7.37 (m, 3H, H-13, 17 & 15), 7.78–7.81 (m, 2H. H-4 & 6). Figure S30: ^13^C NMR (100 MHz, CDCl_3_) δ (ppm): 14.24 (C-29), 43.49 (C-8), 55.56 (C-33), 61.64 (C-28), 63.90 (C-9), 114.48 (C-1 & 3), 117.62 (C-24), 122.15 (C-5), 125.83 (C-4 & 6), 128.53 (C-15), 129.19 (C-13 & 17), 129.36 (C-14 & 16), 139.26 (C-12), 146.94 (C-21), 160.71 (C-7), 162.71 (C-2), 166.69 (C-25), 173.07 (C-20), 178.76 (C-18).

##### Ethyl-2-(2-(3,5-bis(4-methoxyphenyl)-4,5-dihydro-1H-pyrazol-1-yl)-4-oxothiazol-5(4H)-ylidene)acetate (**7k**)


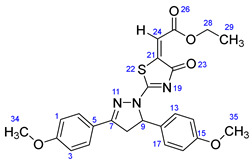


Yellow solid, isolated yield: 86%; mp: 208–210 °C. Figure S31: IR (KBr) ν (cm^−1^): 2937, 2835 (C-H, alkane, stretching), 1693 (C=O, ester), 1609 (C=C, alkene), 1594, 1553 (C=C, aromatic), 1512, 1464 (C=N, stretching), 1311, 1248, 1222 (C-O, stretching), 1174, 1111, 1025 (C-N stretching and aromatic C-H out of plane bending), 862, 839, 809, 768, 728 (aromatic C-H out of plane bending). Figure S32: ^1^H NMR (400 MHz, CDCl_3_) δ (ppm): 1.36 (t, *J =* 8 Hz, 3H, CH_3_-29), 3.42 (dd, *J =* 20, 4 Hz, 1H, H-8_A_), 3.79 (s, 3H, OCH_3_-34), 3.91 (s, 3H, OCH_3_-35), 3.96 (m, 1H, H-8_B_), 4.33 (q, *J =* 8 Hz, 2H, CH_2_-28), 5.81 (dd, *J =* 12, 4 Hz, 1H, H-9), 6.84–6.87 (m, 2H, H-14 & 16), 6.90 (s, 1H, H-24), 6.98–7.02 (m, 2H, H-1 & 3), 7.20–7.24 (m, 2H, H-13 & 17), 7.78–7.82 (m, 2H, H-4 & 6). Figure S33: ^13^C NMR (100 MHz, CDCl_3_) δ (ppm): 14.24 (C-29), 43.33 (C-8), 55.32 (C-35), 55.56 (C-34), 61.62 (C-28), 63.53 (C-9), 114.46 (C-1, 3, 14 & 16), 117.48 (C-24), 122.22 (C-5), 127.44 (C-4 & 6), 129.35 (C-13 & 17), 131.35 (C-12), 146.98 (C-21), 159.66 (C-7), 160.82 (C-15), 162.69 (C-2), 166.71 (C-25), 172.91 (C-20), 178.81 (C-18).

##### Ethyl-2-(2-(5-(4-chlorophenyl)-3-phenyl-4,5-dihydro-1H-pyrazol-1-yl)-4-oxothiazol-5(4H)-ylidene)acetate (**7l**)


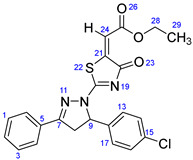


Yellow solid, isolated yield: 82%; mp: 251–253 °C. Figure S34: IR (KBr) ν (cm^−1^): 2987, 2936 (C-H, alkane, stretching), 1684 (C=O, ester), 1601 (C=C, alkene), 1549, 1495 (C=C, aromatic), 1447 (C=N, stretching), 1227 (C-O, stretching), 1179, 1110, 1094, 1021 (C-N stretching and aromatic C-H out of plane bending), 862 (C-Cl, stretching), 829, 765, 727 (aromatic C-H out of plane bending). Figure S35: ^1^H NMR (400 MHz, CDCl_3_) δ (ppm): 1.37 (t, *J* = 8 Hz, 3H, CH_3_-29), 3.43 (dd, *J* = 20, 4 Hz, 1H, H-8_A_), 4.03 (dd, *J* = 18, 12 Hz, 1H, H-8_B_), 4.34 (q, *J* = 8 Hz, 2H, CH_2_-28), 5.87 (dd, *J* = 12, 4 Hz, 1H, H-9), 6.93 (s, 1H, H-24), 7.21–7.24 (m, 2H, H-1 & 3), 7.31–7.35 (m, 2H, H-13 & 17), 7.49–7.59 (m, 3H, H-14, 16 & 2), 7.84–7.86 (m, 2H, H-4 & 6). Figure S36: ^13^C NMR (100 MHz, CDCl_3_) δ (ppm): 14.23 (C-29), 43.35 (C-8), 61.75 (C-28), 63.33 (C-9), 118.16 (C-24), 127.39 (C-4 & 6), 127.52 (C-1 & 3), 129.11 (C-14 & 16), 129.44 (C-2, 13 & 17), 132.15 (C-5), 134.53 (C-15), 137.63 (C-12), 146.52 (C-21), 160.80 (C-7), 166.59 (C-25), 173.81 (C-20), 178.69 (C-18).

##### Ethyl-2-(2-(5-(4-chlorophenyl)-3-(4-methoxyphenyl)-4,5-dihydro-1H-pyrazol-1-yl)-4-oxothiazol-5(4H)-ylidene)acetate (**7m**)


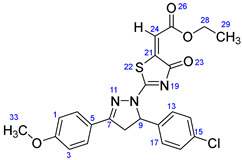


Yellow solid, isolated yield: 92%; mp: 220–222 °C. Figure S37: IR (KBr) ν (cm^−1^): 2971 (C-H, alkane, stretching), 1688 (C=O, ester), 1607 (C=C, alkene), 1589, 1542 (C=C, aromatic), 1513, 1493 (C=N, stretching), 1248 (C-O, stretching), 1177, 1106, 1087, 1031 (C-N stretching and aromatic C-H out of plane bending), 865 (C-Cl, stretching), 844, 814, 795, 768 (aromatic C-H out of plane bending). Figure S38: ^1^H NMR (400 MHz, CDCl_3_) δ (ppm): 1.37 (t, *J =* 8 Hz, 3H, CH_3_-29), 3.38 (dd, *J =* 16, 4 Hz, 1H, H-8_A_), 3.91 (s, 3H, OCH_3_-33), 3.99 (dd, *J =* 18, 12 Hz, 1H, H-8_B_), 4.33 (q, *J =* 8 Hz, 2H, CH_2_-28), 5.84 (dd, *J =* 8, 4 Hz, 1H, H-9), 6.91 (s, 1H, H-24), 6.98–7.02 (m, 2H, H-1 & 3), 7.20–7.23 (m, 2H, H-13 & 17), 7.30–7.33 (m, 2H, H-14 & 16), 7.77–7.81 (m, 2H. H-4 & 6). Figure S39: ^13^C NMR (100 MHz, CDCl_3_) δ (ppm): 14.23 (C-29), 43.34 (C-8), 55.57 (C-33), 61.69 (C-28), 63.24 (C-9), 114.52 (C-1 & 3), 117.85 (C-24), 121.97 (C-5), 127.40 (C-4 & 6), 129.37 (C-14 & 16), 129.39 (C-13 & 17), 134.44 (C-15), 137.74 (C-12), 146.72 (C-21), 160.54 (C-7), 162.80 (C-2), 166.64 (C-25), 173.17 (C-20), 178.66 (C-18).

##### Ethyl-2-(2-(3,5-diphenyl-4,5-dihydro-1H-pyrazol-1-yl)-4-oxothiazol-5(4H)-ylidene)acetate (**7n**)


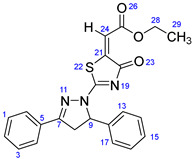


Yellow solid, isolated yield: 80%; mp: 216–218 °C. Figure S40: IR (KBr) ν (cm^−1^): 2985, 2928 (C-H, alkane, stretching), 1693 (C=O, ester), 1602 (C=C, alkene), 1540, 1495 (C=C, aromatic), 1455 (C=N, stretching), 1223 (C-O, stretching), 1185, 1170, 1112, 1033 (C-N stretching and aromatic C-H out of plane bending), 858, 825, 765, 742 (aromatic C-H out of plane bending). Figure S41: ^1^H NMR (400 MHz, CDCl_3_) δ (ppm): 1.37 (t, *J =* 8 Hz, 3H, CH_3_-29), 3.46 (dd, *J =* 16, 4 Hz, 1H, H-8_A_), 4.02 (dd, *J =* 16, 12 Hz, 1H, H-8_B_), 4.34 (q, *J =* 8 Hz, 2H, CH_2_-28), 5.90 (dd, *J =* 12, 4 Hz, 1H, H-9), 6.93 (s, 1H, H-24), 7.28–7.38 (m, 5H, H-13, 14, 15, 16 & 17), 7.48–7.58 (m, 3H, H-1, 2 & 3), 7.85–7.87 (m, 2H, H-4 & 6). Figure S42: ^13^C NMR (100 MHz, CDCl_3_) δ (ppm): 14.23 (C-29), 43.51 (C-8), 61.70 (C-28), 63.99 (C-9), 117.94 (C-24), 125.82 (C-4 & 6), 127.53 (C-13 & 17), 128.60 (C-15), 129.06 (C-1 & 3), 129.24 (C-14 & 16), 129.62 (C-2), 132.04 (C-5), 139.14 (C-12), 146.72 (C-21), 161.00 (C-7), 166.63 (C-25), 173.71 (C-20), 178.79 (C-18).

### 3.3. Biology

#### 3.3.1. Alkaline Phosphatase Inhibition Assay

As previously mentioned [65,68], spectrophotometric analysis was used to evaluate the activity of calf intestinal alkaline phosphatase (CIAP). The reaction mixture contained 50 mM Tris-HCl buffer (5 mM MgCl_2_, 0.1 mM ZnCl_2_ pH 9.5), the 10 µL compound, and it was preincubated for 10 min by adding 5 µL of CIAP (0.025 U/mL). Then, 10 µL of a substrate (0.5 mM *p*-NPP (para nitrophenyl phosphate disodium salt)) was delivered to initiate the response, and therefore the assay mixture became incubated once more for 30 min at 37 °C. The change in absorbance of released *p*-nitrophenolate was monitored at 405 nm, using a 96-well microplate reader (Thermo Scientific Multiskan GO, USA). All the experiments were repeated three times in a triplicate manner. KH_2_PO_4_ (potassium dihydrogen phosphate) was used as the reference inhibitor of CIAP. The inhibition activity was calculated according to the following equation:

Inhibition (%)=[(Blank−Sample)/Blank]×100

For calculation of the IC_50_ values, 6–8 serial dilutions were used and the results examined using the non-linear regression analysis of GraphPad Prism 5 [69].

#### 3.3.2. Kinetic Mechanism Analysis

Primarily based on the IC_50_ findings, we selected the most effective inhibitor **7g** for CIAP to determine the mechanism of enzyme inhibition using our published method [70]. The inhibitor (**7g**), and substrate *p*-NPP concentrations were used at 0.00, 0.023, 0.045 and 0.091 µM, 10, 5, 2.5, 1.25, 0.625 and 0.3125 mM, respectively. The pre-incubation time and other conditions were the same as those mentioned in the section on the CIAP assay. Maximal initial velocities were determined from the initial linear portion of absorbances up to 10 min after the addition of enzyme per minute interval. The inhibition type of the enzyme was assayed by the Lineweaver–Burk plot of inverse velocities (1/V) versus the inverse of substrate concentration 1/[S] mM^−1^. The EI dissociation constant *K*i was determined by a secondary plot of 1/V versus inhibitor concentration.

#### 3.3.3. Free Radical Scavenging Assay

Free radical scavenging activity was determined by modifying the method by DPPH assay [71]. The assay solution composed of the 100 μL of DPPH (150 μM) and 20 μL of the sample (100 μg/mL) was introduced to gain the final volume of each well up to 200 μL with methyl alcohol. Then, it was incubated in the dark at room temperature for the following half-hour. Ascorbic acid (Vitamin C) was used as a reference inhibitor. The measurements were performed by using a microplate reader (Thermo Scientific Multiskan GO, USA) at 517 nm. Each experiment was carried out in triplicate.

#### 3.3.4. Cell Culture and Treatment of Compounds

The human osteosarcoma MG-63 cells were acquired from the Korean Cell Line Bank (Seoul, Korea). The MG-63 cell lines were incubated in the humidified atmosphere at 37 °C containing 5% CO_2_ and maintained in the DMEM (Sigma, St. Louis, MO, USA), supplemented with 10% fetal bovine serum (FBS, WELGENE, Korea) and 50 unit/mL penicillin (Sigma) and 50 μg/mL streptomycin (Sigma). The medium was completely renewed every two days. The synthesized compounds were completely dissolved in dimethyl sulfoxide (DMSO) and further diluted to various concentrations (0, 0.5, 1, 2, and 4 µM). The control groups were treated with the same amount of DMSO.

#### 3.3.5. Cell Viability

MTT assay technique was applied to check the toxic effect of our compounds. The MG-63 cell lines were seeded onto 96-well plates at a density of 0.4 × 10^5^ cells/well with the medium for 24 h. After reaching 50% density, the cells were exposed to synthesized target compounds at the dose of 0, 0.5, 1, 2, and 4 µM for 24 h in media. We employed erlotinib as a positive control to compare the results of the synthesized derivatives. After 24 h, the cell sustained 100 μL DMEM containing of 0.5 mg/mL 3-(4,5-dimethylthiazol-2-yl)-2,5-diphenyltetrazolium bromide (MTT) at 37 °C for 4 h. Then, the purple formazan crystals were dissolved with 100 μL of buffer (10% SDS, 0.01 N HCl). Finally, the absorbance was measured at 570 nm using a microplate reader (Thermo Fisher, Waltham, MA, USA).

### 3.4. Computational Methodology

#### 3.4.1. Chemoinformatic Analysis of Designed Ligands

The synthesized ligands (**7a**–**7n**) were sketched in ACD/ChemSketch and further minimized by UCSF Chimera 1.10.1., whereas biochemical properties and Lipinski’s rule of five (RO5) of synthesized compounds were predicted and justified, respectively, using online computational tools such as Molinspiration (http://www.molinspiration.com/) and Molsoft (http://www.molsoft.com/ accessed on the 8 September 2022).

#### 3.4.2. Retrieval of Alkaline Phosphate Structure from PDB

The three-dimensional (3D) structure of alkaline phosphate from the human placenta was accessed from Protein Data Bank (PDB) (www.rcsb.org) having PDBID 1EW2. The selected target protein structure was minimized by using the UCSF Chimera 1.10.1 tool [72,73]. The protein architecture and statistical percentage values of receptor proteins helices, β-sheets, coils, and turn were predicted from the online server VADAR 1.8 [74].

#### 3.4.3. Designing of Ligands and Molecular Docking

The synthesized ligands were sketched in the drawing ACD/Chem Sketch tool and minimized by UCSF Chimera 1.10.1. All the synthesized ligands were sketched in ACD/Chem Sketch tool and accessed in mol format. Furthermore, the UCSF Chimera 1.10.1 tool was employed for energy minimization of each ligand separately having default parameters such as steepest descent steps 100 with step size 0.02 (Å), conjugate gradient steps 100 with step size 0.02 (Å), and update interval was fixed at 10. Finally, Gasteiger charges were added using Dock Prep in ligand structure to obtain the good structure conformation. The molecular docking experiment was used on all the synthesized ligands against ALP by using the PyRx 0.8, virtual screening tool with Auto Dock VINA Wizard v.1.2.0. approach [75]. The grid box center values of (center X = 43.3, center Y = 23.1612, and center Z = 9.1269), and size values were adjusted as (X = 65.56, Y = 71.79, and Z = 64.64) for better conformational position in the active region of the target protein. All the synthesized ligands were docked separately against ALP with a default exhaustiveness value = 8. The predicted docked complexes were evaluated based on the lowest binding energy (kcal/mol) values and structure activity relationship (SAR). The three-dimensional graphical depictions of all the docked complexes were accomplished by Discovery Studio (2.1.0) and UCSF Chimera 1.10.1 tool.

## 4. Conclusions

In the current study, we have successfully synthesized and characterized fourteen pyrazolo-oxothiazolidine derivatives using a multistep synthetic process. All the synthesized compounds were evaluated for in vitro alkaline phosphatase inhibitor activity and found to be stronger than monopotassium phosphate. Among them, compound **7g** (IC_50_ = 0.045 ± 0.004 μM) had the most effective inhibitory effect against alkaline phosphatases compared to the standard reference KH_2_PO_4_ (IC_50_ = 5.242 ± 0.472 μM). Furthermore, the MTT assay was performed against MG-63 at various concentrations to screen the cell viability potential of synthesized compounds, and they showed no toxic effect on the viability of cells. Molecular docking studies were conducted, to determine the binding affinity of the ligands with the target protein within the active site, and all compounds exhibited good docking binding affinity with alkaline phosphatase and showed good docking scores. According to research findings, these compounds, particularly **7g**, might employ as worthwhile therapeutic candidates for alkaline phosphatase-associated disorders in medicinal chemistry.

## Figures and Tables

**Table 1:** Alkaline phosphatase inhibitory activity of pyrazolo-oxothiazolidine derivatives (**7a**–**7n**).

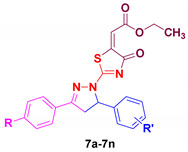				
No.	Compound	R	R′	Alkaline Phosphatase IC_50_ ± SEM (μM)
**1**	7a	H	4-Br	0.053 ± 0.001
**2**	7b	H	4-CH_3_	2.298 ± 0.151
**3**	7c	H	4-OCH_3_	0.198 ± 0.018
**4**	7d	H	3-OCH_3_-4-OCH_2_C_6_H_5_	0.293 ± 0.039
**5**	7e	OCH_3_	4-CH_3_	0.228 ± 0.012
**6**	7f	OCH_3_	2,4-Cl_2_	0.566 ± 0.048
**7**	**7g**	**OCH_3_**	**3-OCH_3_-4-OCH_2_C_6_H_5_**	**0.045 ± 0.004**
**8**	7h	OCH_3_	4-Br	0.907 ± 0.041
**9**	7i	OCH_3_	4-F	0.315 ± 0.017
**10**	7j	OCH_3_	H	0.434 ± 0.011
**11**	7k	OCH_3_	4-OCH_3_	0.075 ± 0.005
**12**	7l	H	4-Cl	0.051 ± 0.019
**13**	7m	OCH_3_	4-Cl	0.302 ± 0.004
**14**	7n	H	H	2.987 ± 0.734
**15**	**KH_2_PO_4_**			**5.242 ± 0.472**

Values are presented as mean ± SEM (Standard error of the mean).

**Table 2:** Kinetic parameters of the compound **7g** for the alkaline phosphatase.

**Concentrations (**µM)	V_max_ (ΔA/Min)	**K_m_** **(mM)**	**Inhibition Type**	***K*i** **(**µM)
0.000	0.0023	0.5	Non-Competitive	0.093
0.023	0.0014	0.5		
0.045	0.0011	0.5		
0.091	0.0009	0.5		

V_max_ = the reaction velocity; K_m_ = Michaelis-Menten constant; *K*i = EI dissociation constant.

**Table 3:** Biological properties of synthesized compounds.

Ligands	Mol. Weight (g/mol)	No. HBA	No. HBD	Mol. Log*P*	MolPSA (A^2^)	Mol. Vol (A^3^)	Drug Likeness Score
**7a**	483	6	0	5.46	56	430	0.09
**7b**	419	6	0	5.07	56	429	−0.01
**7c**	435	7	0	4.58	56	429	0.07
**7d**	541	8	0	5.83	71	545	0.02
**7e**	449	7	0	5.14	63	461	0.00
**7f**	503	7	0	6.14	63	475	0.58
**7g**	571	9	0	5.91	78	577	0.04
**7h**	513	7	0	5.54	63	462	0.14
**7i**	453	7	0	4.76	63	446	0.32
**7j**	435	7	0	4.70	63	440	0.07
**7k**	465	8	0	4.65	71	472	0.07
**7l**	439	6	0	5.22	56	426	0.44
**7m**	469	7	0	5.29	63	457	0.52
**7n**	405	6	0	4.62	56	408	−0.26

**Table 4:** Docking energy values of synthesized ligands against ALP.

Docking Complexes	Docking Energy (Kcal/mol)
ALP_**7a**	−7.8
ALP_**7b**	−7.9
ALP_**7c**	−7.7
ALP_**7d**	−8.3
ALP_**7e**	−7.9
ALP_**7f**	−8
ALP_**7g**	−8.1
ALP_**7h**	−7.5
ALP_**7i**	−7.9
ALP_**7j**	−7.9
ALP_**7k**	−7.7
ALP_**7l**	−7.9
ALP_**7m**	−7.9
ALP_**7n**	−7.9

## Data Availability

The dataset generated during the current study will be available from the corresponding author upon reasonable request.

## Supplementary Materials

The following supporting information can be downloaded at: https://www.mdpi.com/article/10.3390/ijms232113262/s1. Reference [76] are cited in the Supplementary Materials.
