# Supplementary material for: Potent Alkaline Phosphatase Inhibitors, Pyrazolo-Oxothiazolidines: Synthesis, Biological Evaluation, Molecular Docking, and Kinetic Studies
Source: Int J Mol Sci. 2022 Oct 31;23(21):13262. doi: 10.3390/ijms232113262 (PMC9654533; doi:10.3390/ijms232113262)
Supplement: Supplementary file 1 [file ijms-23-13262-s001.zip › ijms-1965757-supplementary.pdf]

International Journal of
Molecular Sciences

MDPI

Supporting Information

Potent Alkaline Phosphatase Inhibitors, Pyrazolo-Oxothiazolidines: Synthesis, Biological Evaluation, Molecular Docking, and Kinetic Studies

Narges Hosseini Nasab [a], Hussain Raza [a], Rok Su Shim [a], Mubashir Hassan [b], Andrzej Kloczkowski [b] and Song Ja Kim [a,*]

[a] Department of Biological Sciences, Kongju National University, Gongju, Chungnam 32588, Korea
[b] The Steve and Cindy Rasmussen Institute for Genomic Medicine at Nationwide Children's Hospital, Department of Pediatrics, Columbus, Ohio 43205, United States of America
* Correspondence: ksj85@kongju.ac.kr

Supporting Figure captions:

Fig. S1: IR Spectrum of 7a

Fig. S2: ^{1}H NMR Spectrum of 7a

Fig. S3: ^{13}C NMR Spectrum of 7a

Fig. S4: IR Spectrum of 7b

Fig. S5: ^{1}H NMR Spectrum of 7b

Fig. S6: ^{13}C NMR Spectrum of 7b

Fig. S7: IR Spectrum of 7c

Fig. S8: ^{1}H NMR Spectrum of 7c

Fig. S9: ^{13}C NMR Spectrum of 7c

Fig. S10: IR Spectrum of 7d

Fig. S11: ^{1}H NMR Spectrum of 7d

Fig. S12: ^{13}C NMR Spectrum of 7d

Fig. S13: IR Spectrum of 7e

Fig. S14: ^{1}H NMR Spectrum of 7e

Fig. S15: ^{13}C NMR Spectrum of 7e

Fig. S16: IR Spectrum of 7f

Fig. S17: ^{1}H NMR Spectrum of 7f

Fig. S18: ^{13}C NMR Spectrum of 7f

Fig. S19: IR Spectrum of 7g

Fig. S20: ^{1}H NMR Spectrum of 7g

Fig. S21: ^{13}C NMR Spectrum of 7g

Fig. S22: IR Spectrum of 7h

Fig. S23: ^{1}H NMR Spectrum of 7h

Fig. S24: ^{13}C NMR Spectrum of 7h

Fig. S25: IR Spectrum of 7i

Fig. S26: ^{1}H NMR Spectrum of 7i

Fig. S27: ^{13}C NMR Spectrum of 7i

Fig. S28: IR Spectrum of 7j

Fig. S29: ^{1}H NMR Spectrum of 7j

Fig. S30: ^{13}C NMR Spectrum of 7j

Fig. S31: IR Spectrum of 7k

Fig. S32: ^{1}H NMR Spectrum of 7k

Fig. S33: ^{13}C NMR Spectrum of 7k

Fig. S34: IR Spectrum of 7l

Fig. S35: ^{1}H NMR Spectrum of 7l

Fig. S36: ^{13}C NMR Spectrum of 7l

Fig. S37: IR Spectrum of 7m

Fig. S38: ^{1}H NMR Spectrum of 7m

Fig. S39: ^{13}C NMR Spectrum of 7m

Fig. S40: IR Spectrum of 7n

Fig. S41: ^{1}H NMR Spectrum of 7n

Fig. S42: ^{13}C NMR Spectrum of 7n

Fig. S43: 2D-docking depiction of 7a complex against alkaline phosphatase

Fig. S44: 2D-docking depiction of 7b complex against alkaline phosphatase

Fig. S45: 2D-docking depiction of 7c complex against alkaline phosphatase

Fig. S46: 2D-docking depiction of 7d complex against alkaline phosphatase

Fig. S47: 2D-docking depiction of 7e complex against alkaline phosphatase

Fig. S48: 2D-docking depiction of 7f complex against alkaline phosphatase

Fig. S49: 2D-docking depiction of 7g complex against alkaline phosphatase

Fig. S50: 2D-docking depiction of 7h complex against alkaline phosphatase

Fig. S51: 2D-docking depiction of 7i complex against alkaline phosphatase

Fig. S52: 2D-docking depiction of 7j complex against alkaline phosphatase

Fig. S53: 2D-docking depiction of 7k complex against alkaline phosphatase

Fig. S54: 2D-docking depiction of 7l complex against alkaline phosphatase

Fig. S55: 2D-docking depiction of 7m complex against alkaline phosphatase

Fig. S56: 2D-docking depiction of 7n complex against alkaline phosphatase

The detection of signals originating from common contaminants during routine NMR use presents a challenge (water, solvents, stabilizers, oils). Grease (silicon grease) is a typical contaminant used in glassware joints. Grease peaks in $CDCl_3$ as a solvent can be found at δ 0.07, 0.08, and 1.26 ppm in ^{1}H NMR and at δ 1.04 and 29.76 ppm in ^{13}C NMR [1].

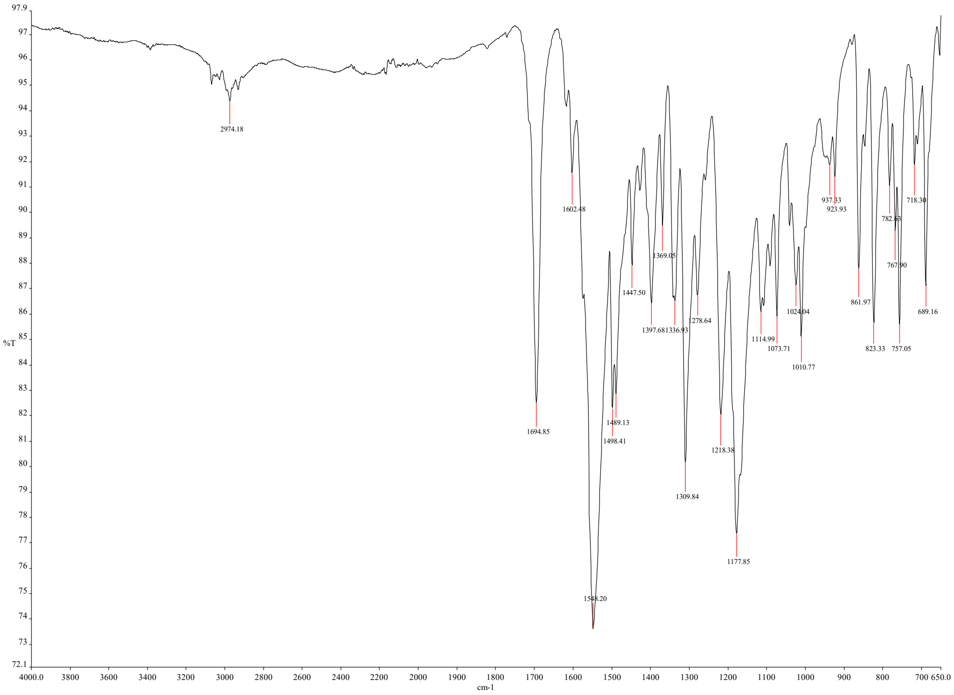

Fig. S1, ES‡

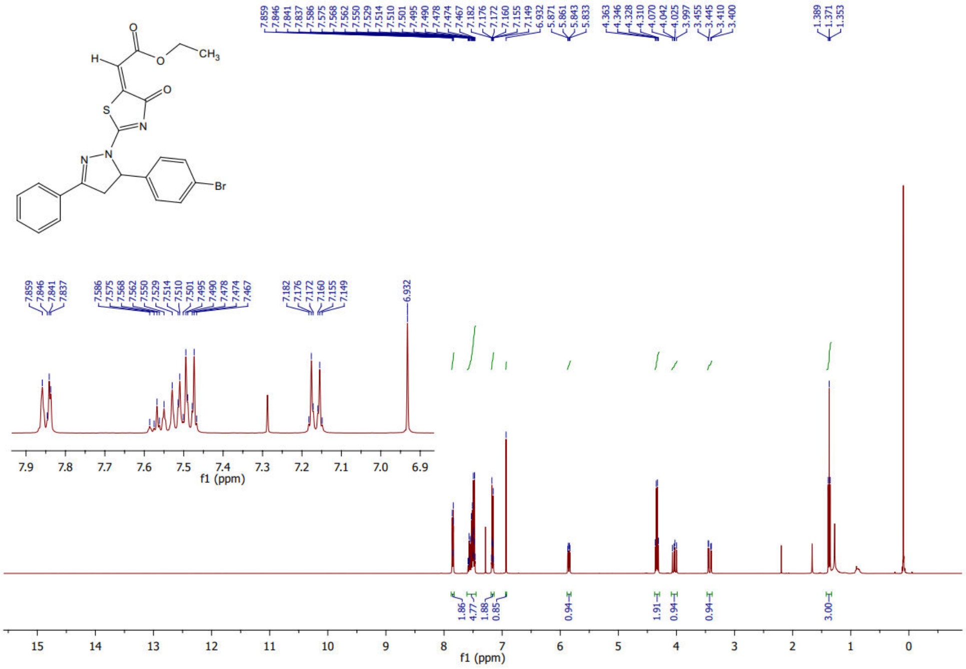

Fig. S2, ES‡

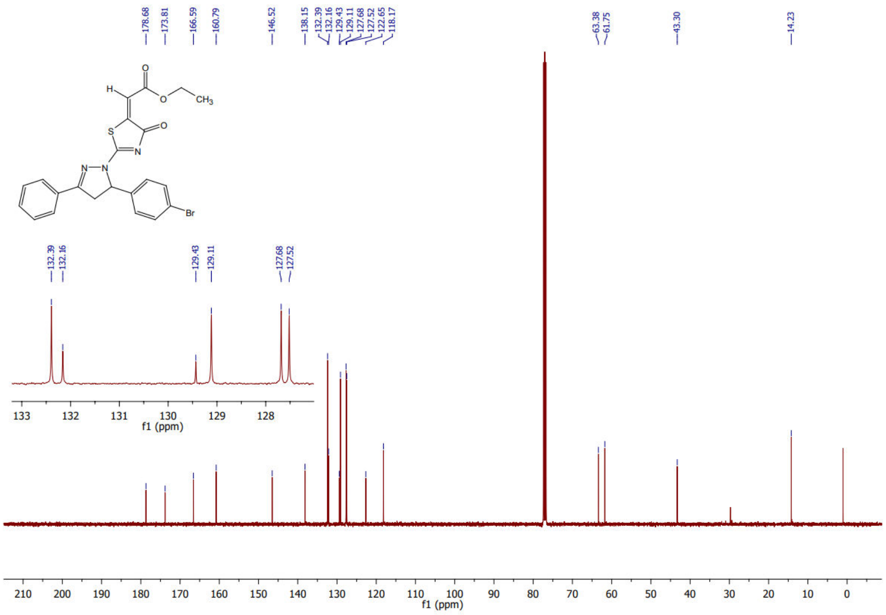

Fig. S3, ES‡

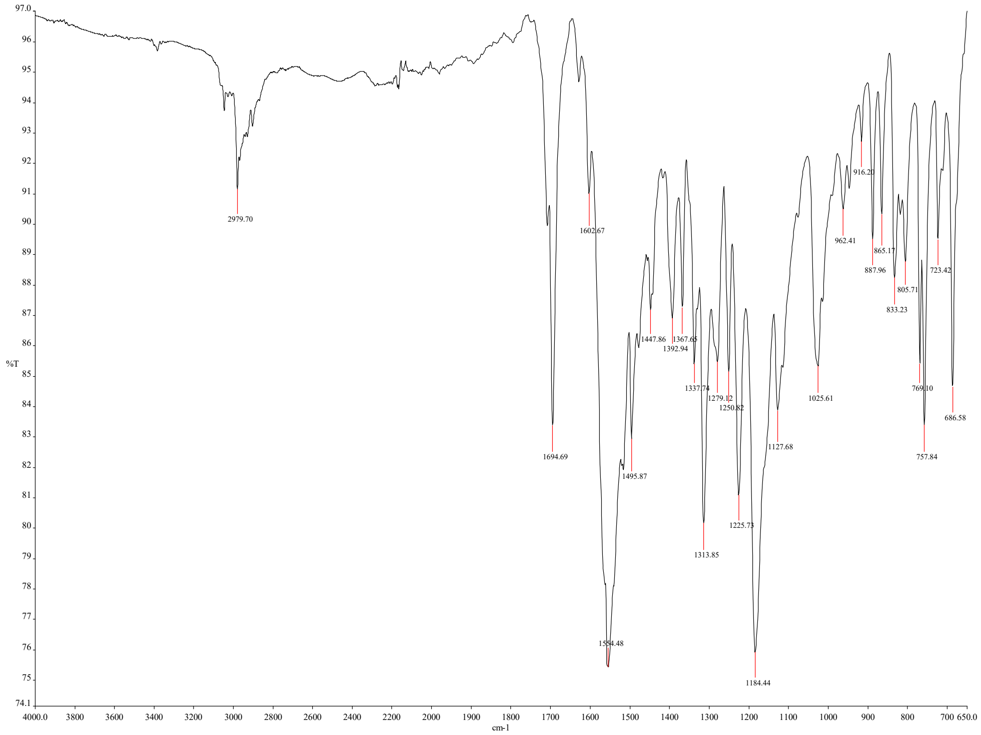

Fig. S4, ES‡

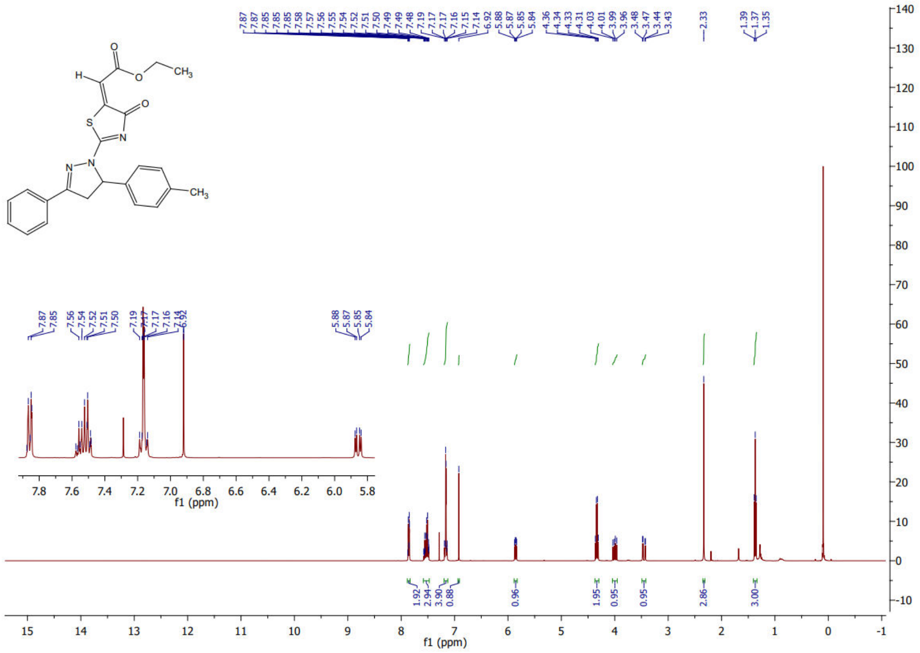

Fig. S5, ES‡

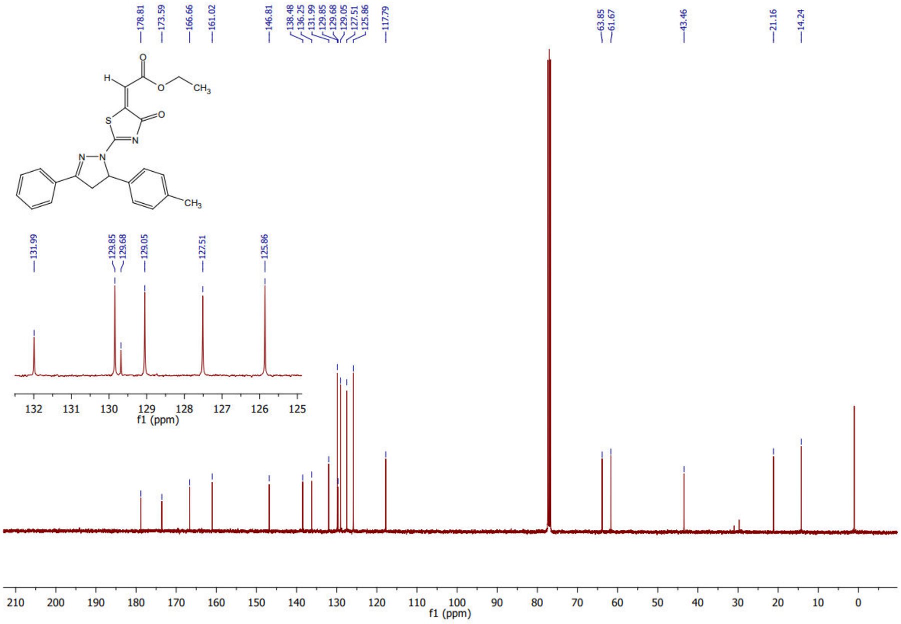

Fig. S6, ES‡

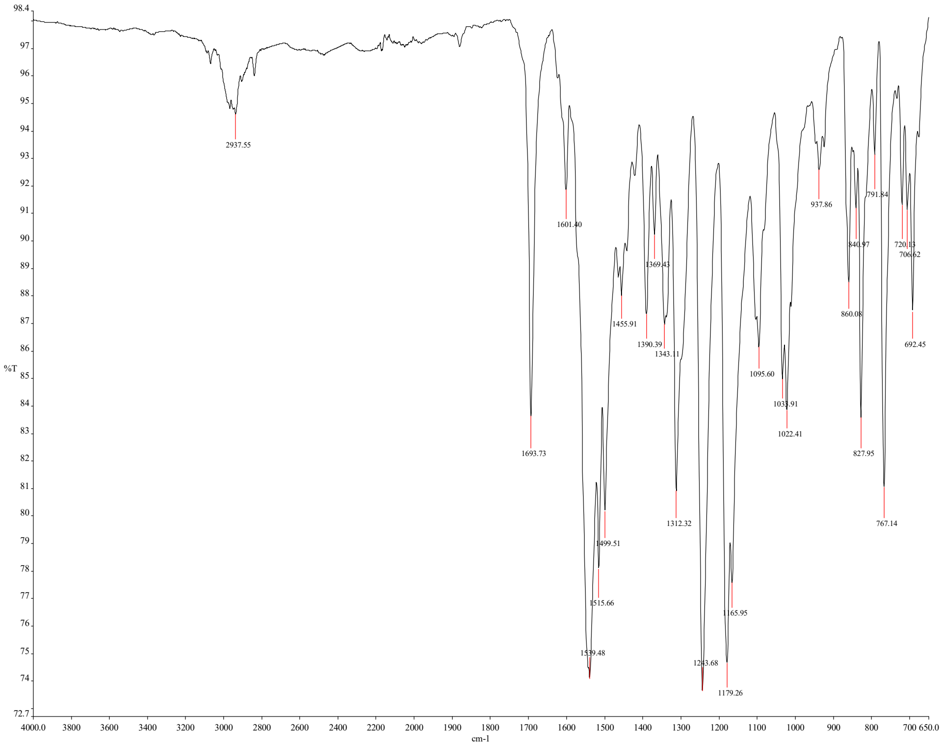

Fig. S7, ES‡

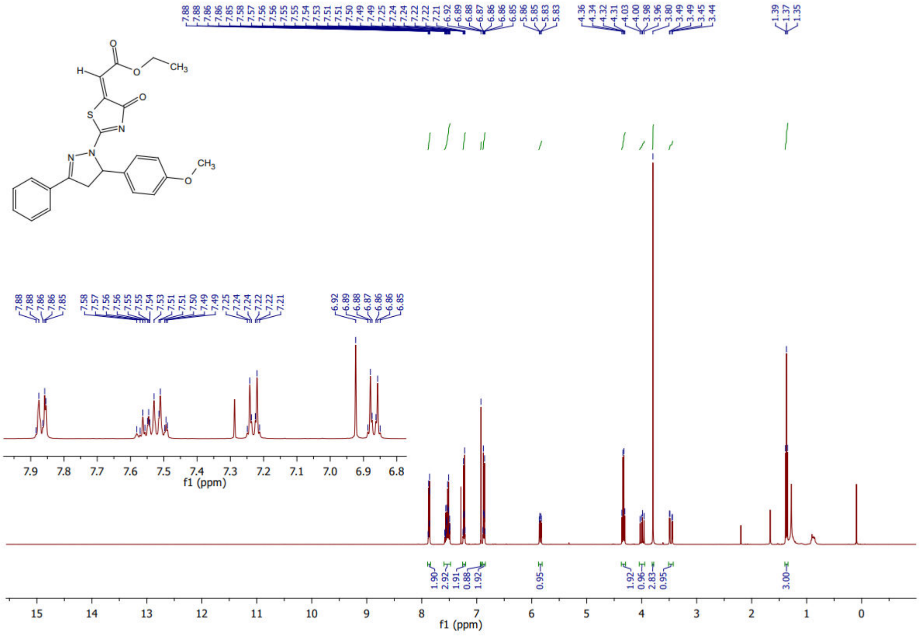

Fig. S8, ES‡

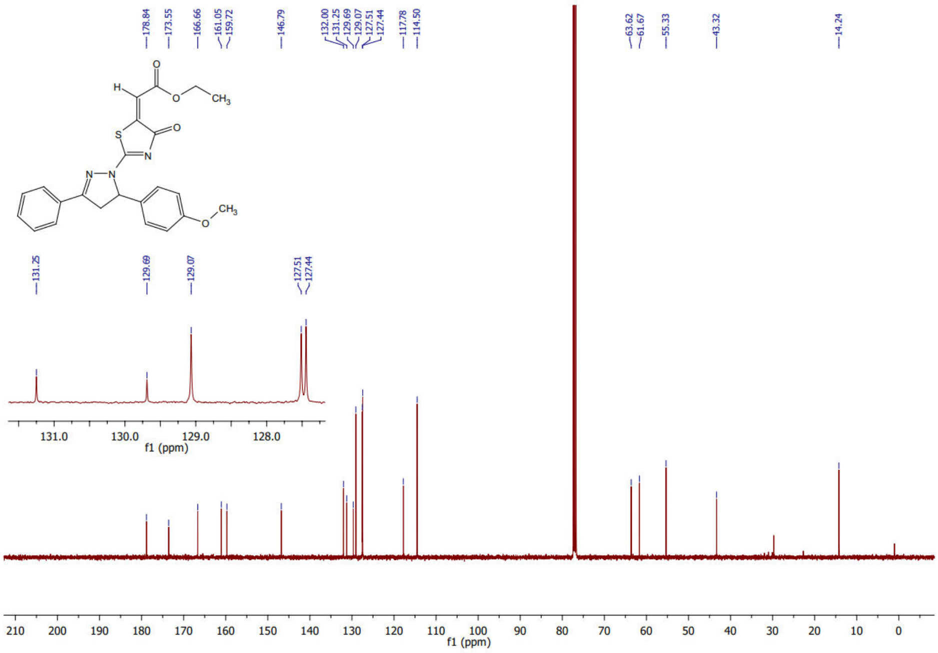

Fig. S9, ES‡

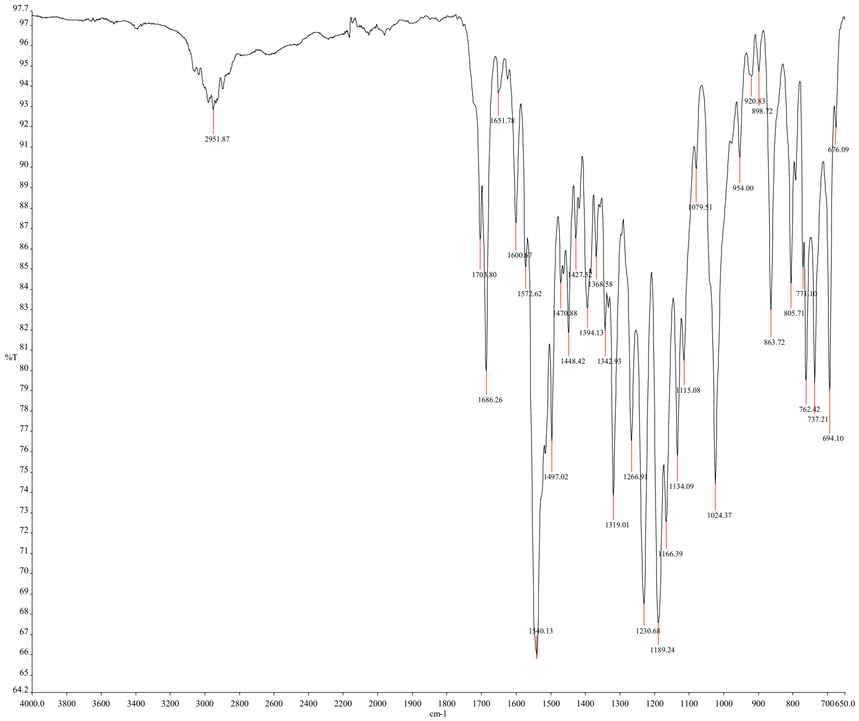

Fig. S10, ES‡

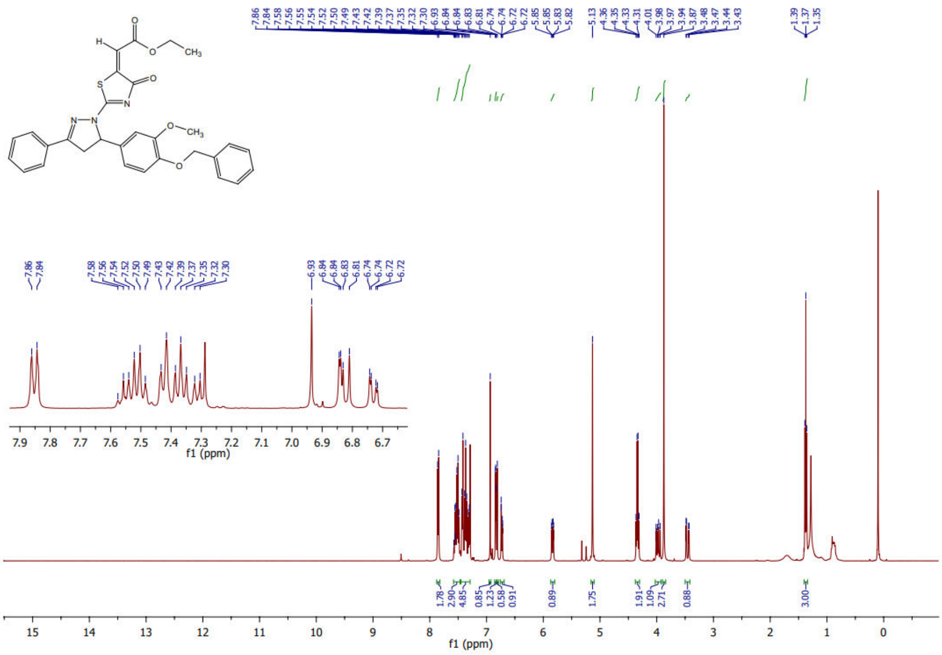

Fig. S11, ES‡

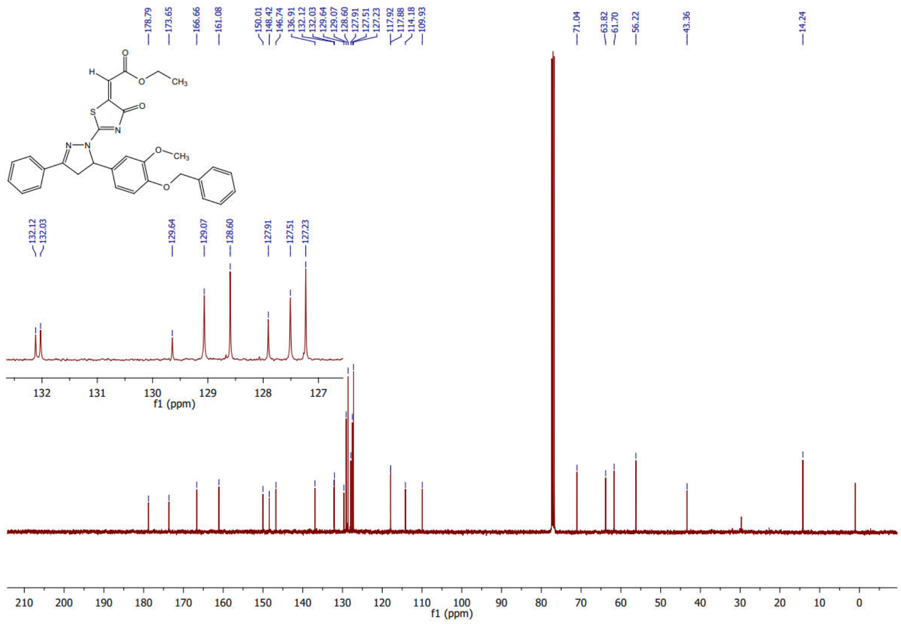

Fig. S12, ES‡

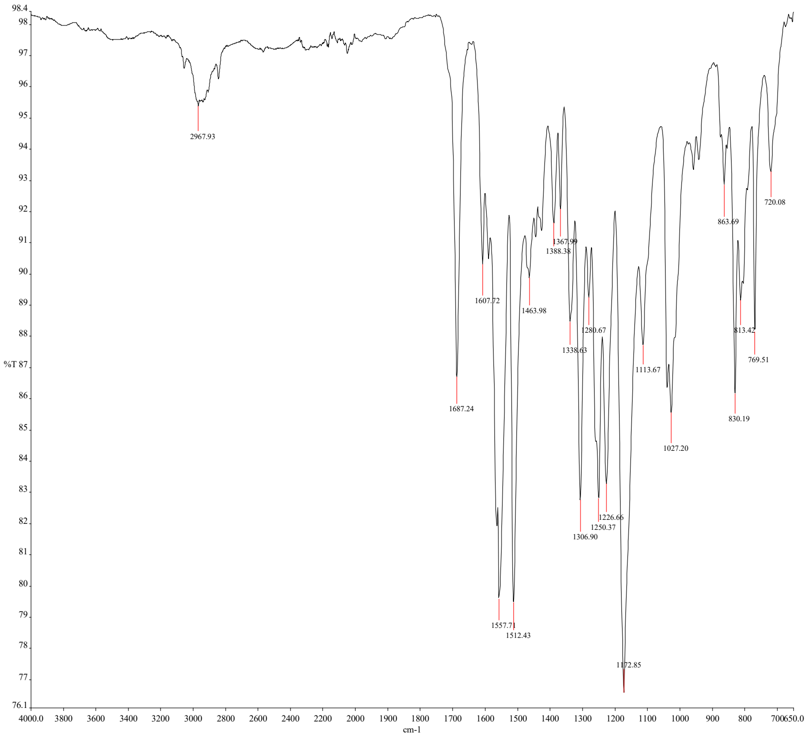

Fig. S13, ES‡

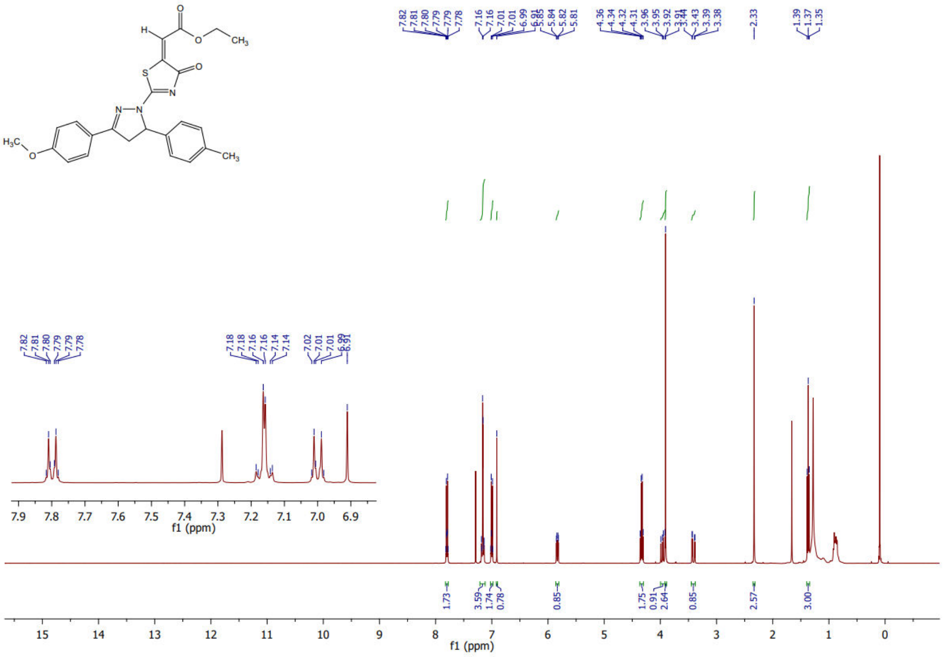

Fig. S14, ES‡

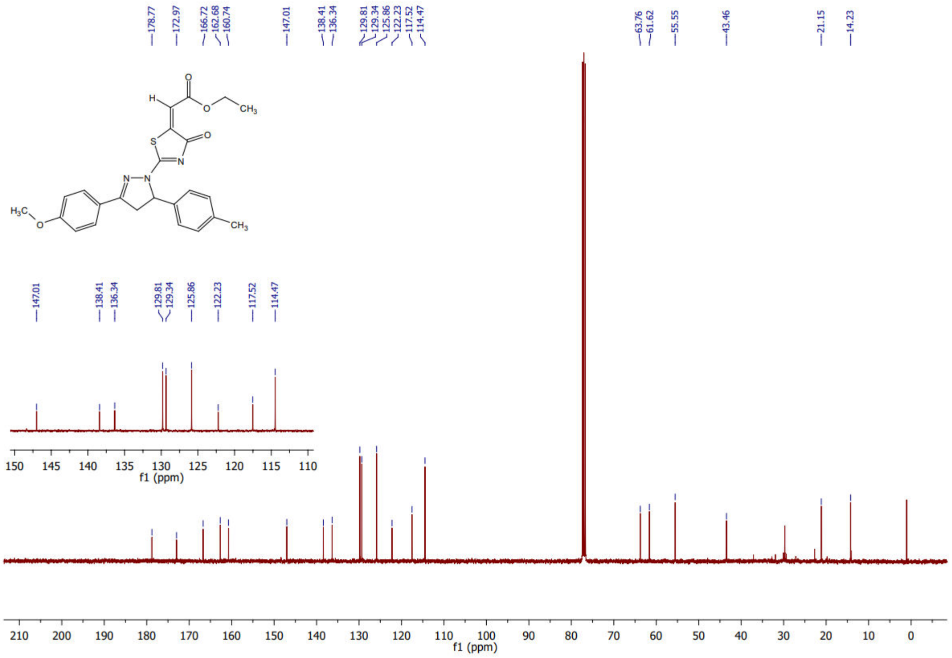

Fig. S15, ES‡

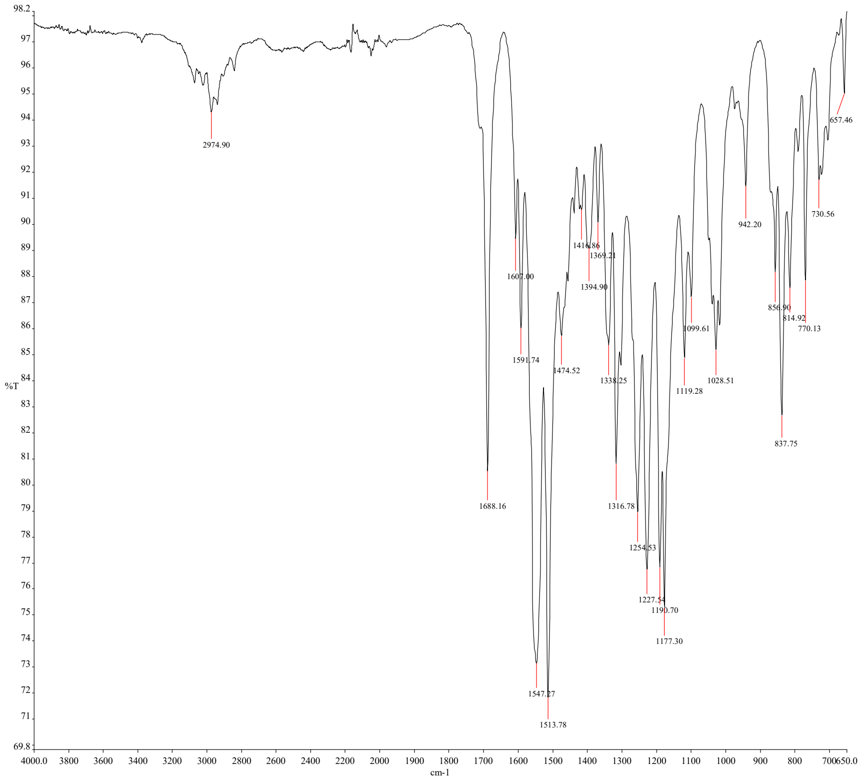

Fig. S16, ES‡

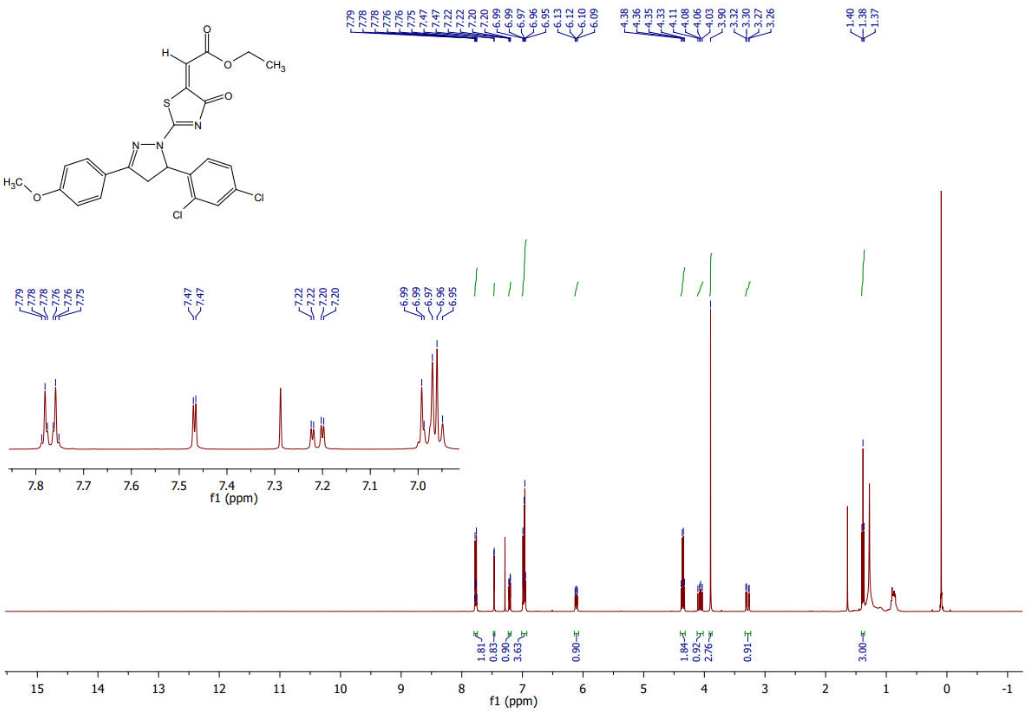

Fig. S17, ES‡

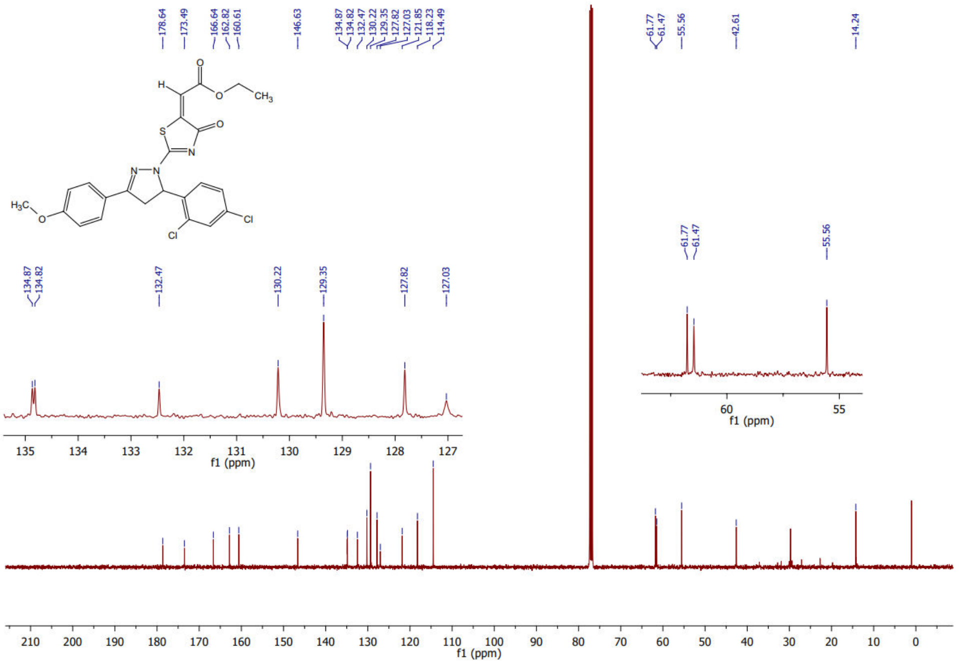

Fig. S18, ES‡

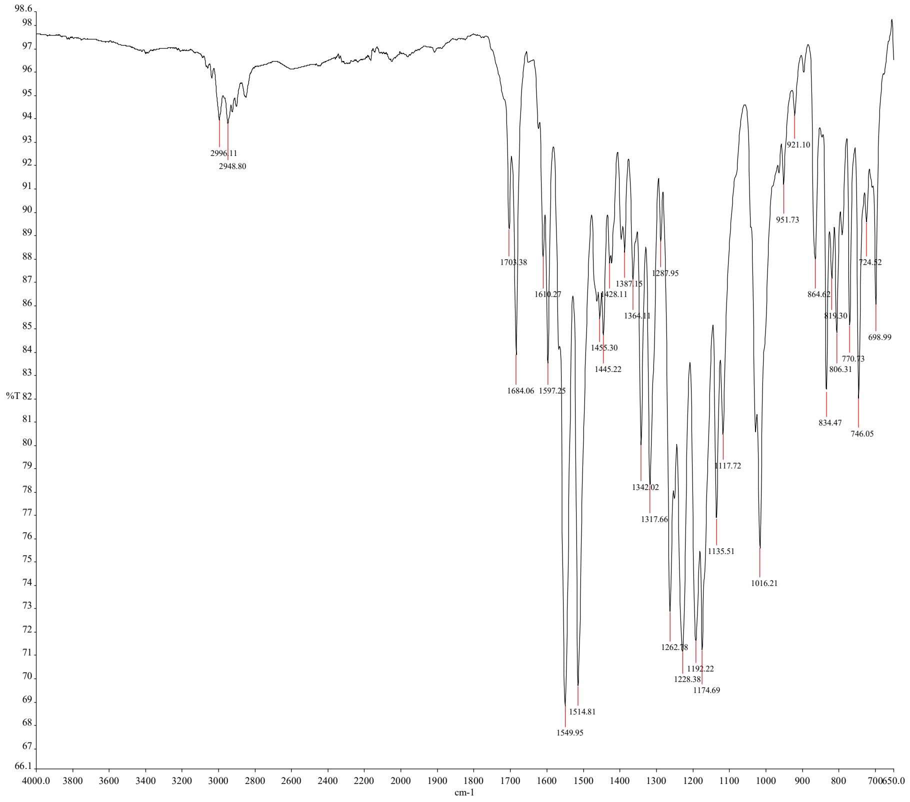

Fig. S19, ES‡

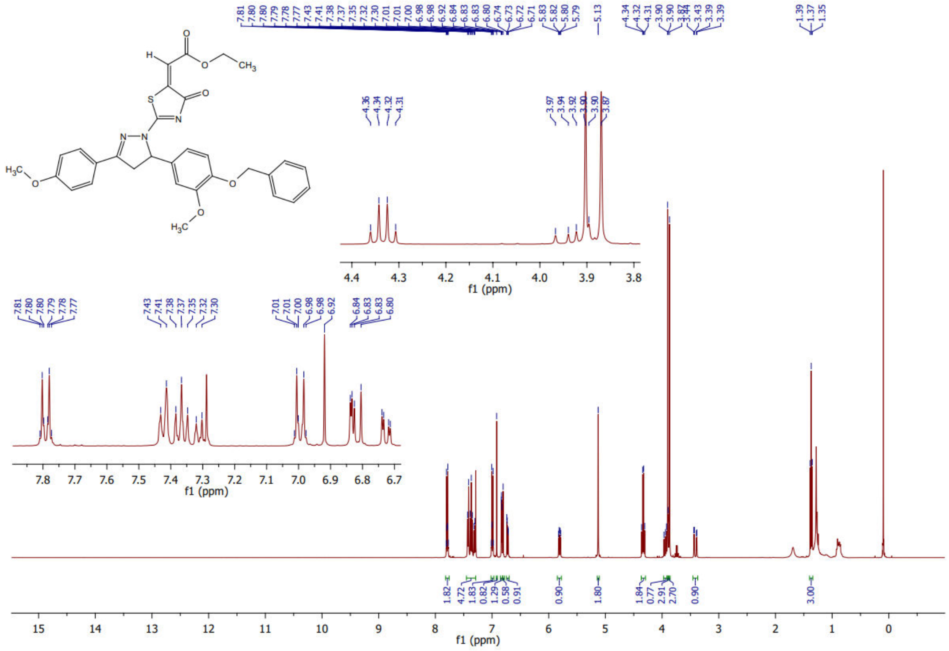

Fig. S20, ES‡

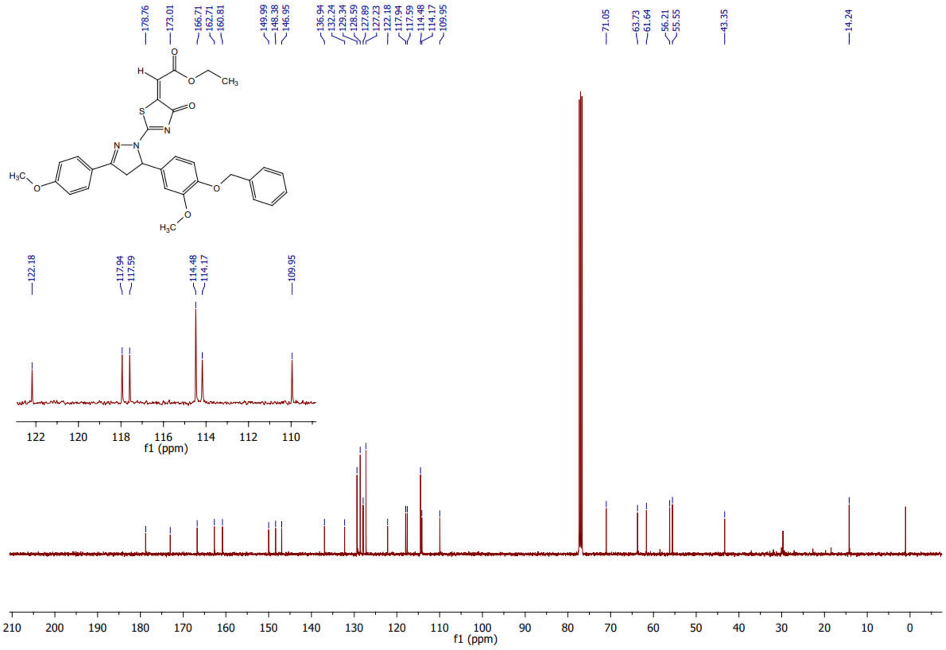

Fig. S21, ES‡

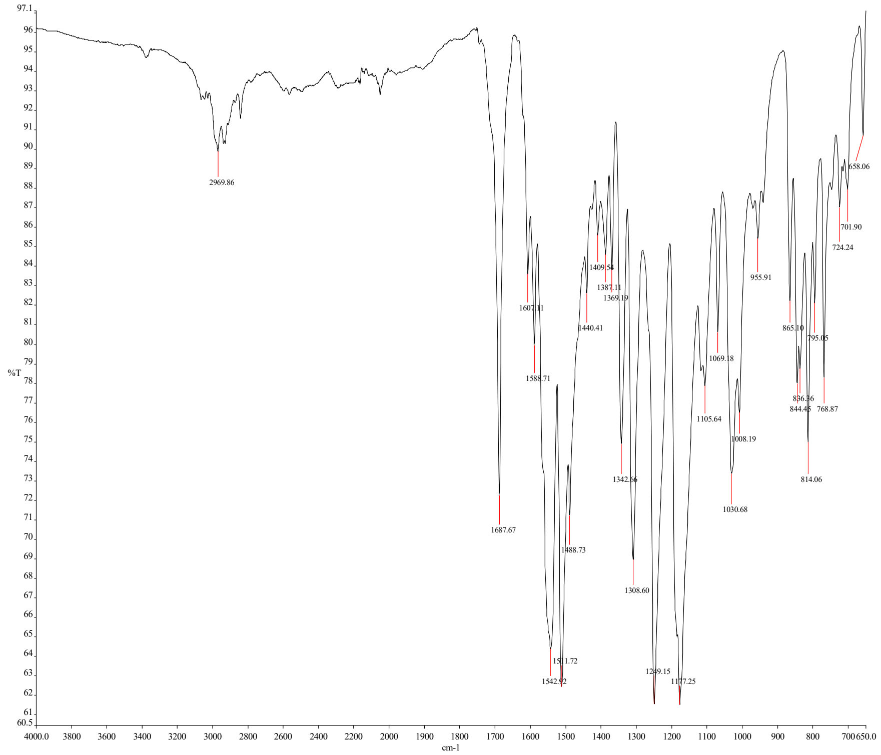

Fig. S22, ES‡

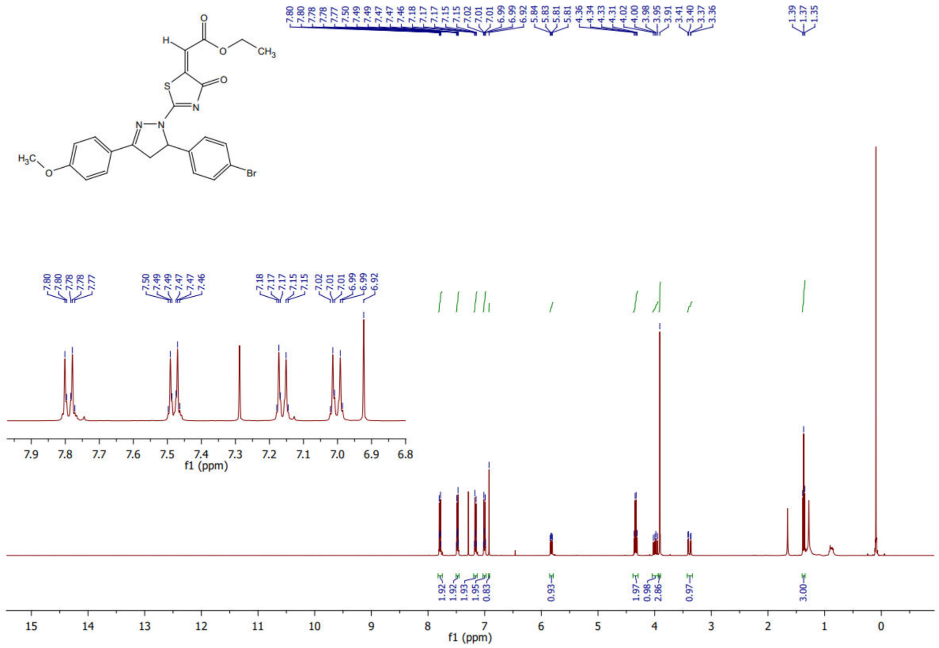

Fig. S23, ES‡

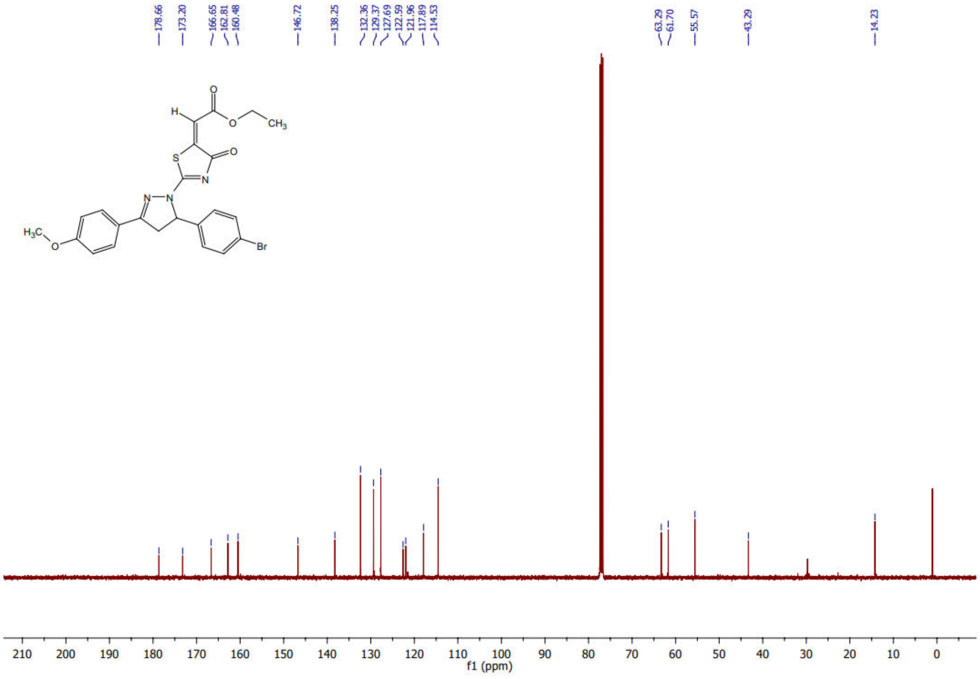

Fig. S24, ES‡

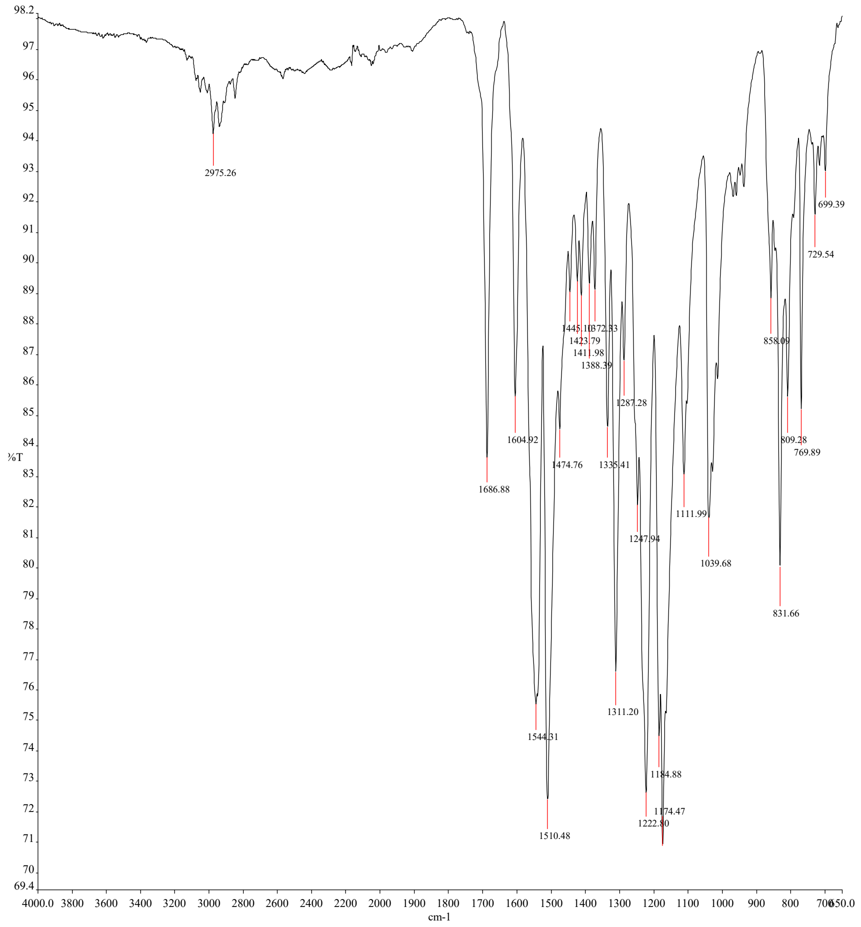

Fig. S25, ES‡

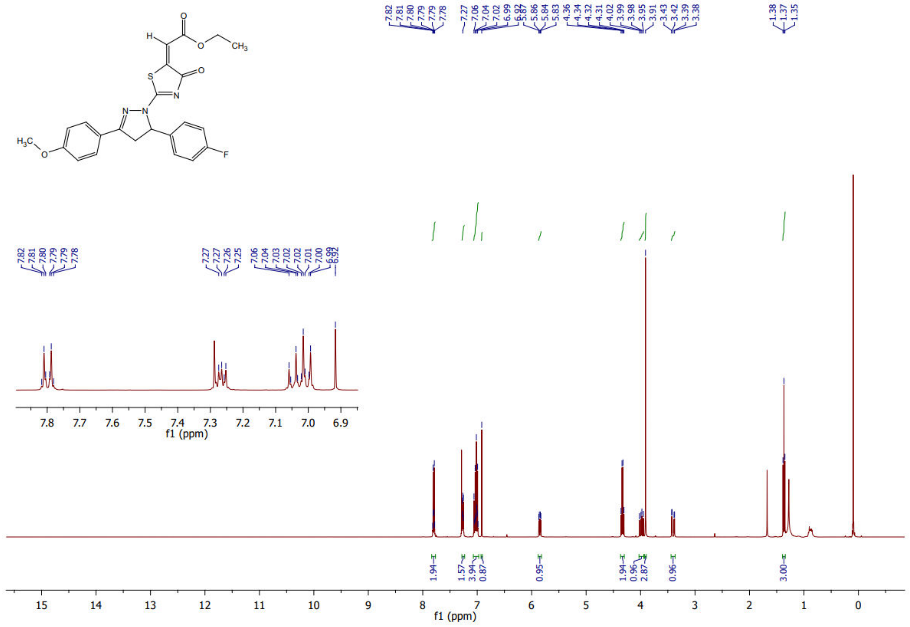

Fig. S26, ES‡

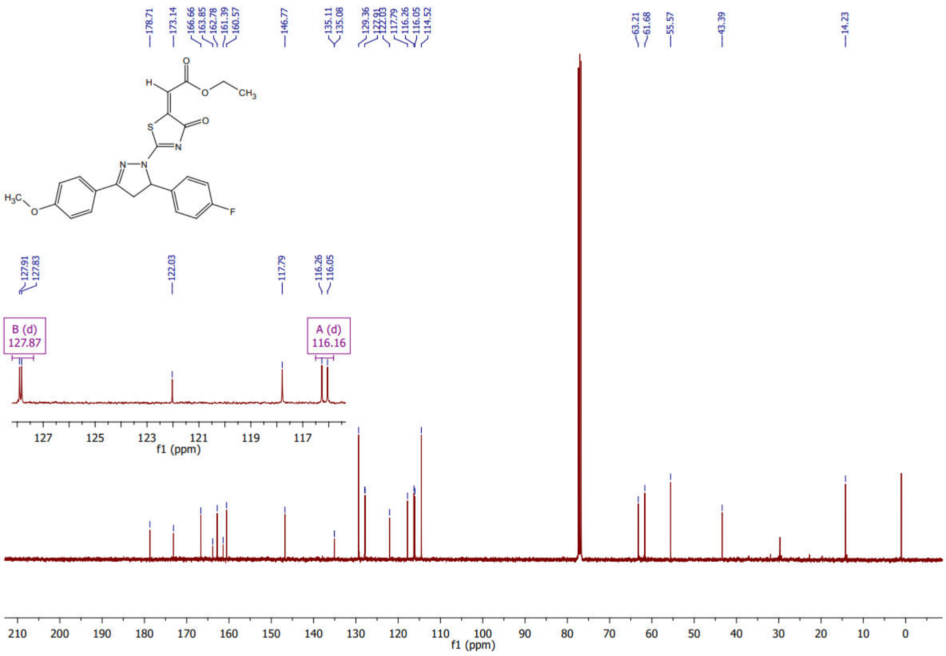

Fig. S27, ES‡

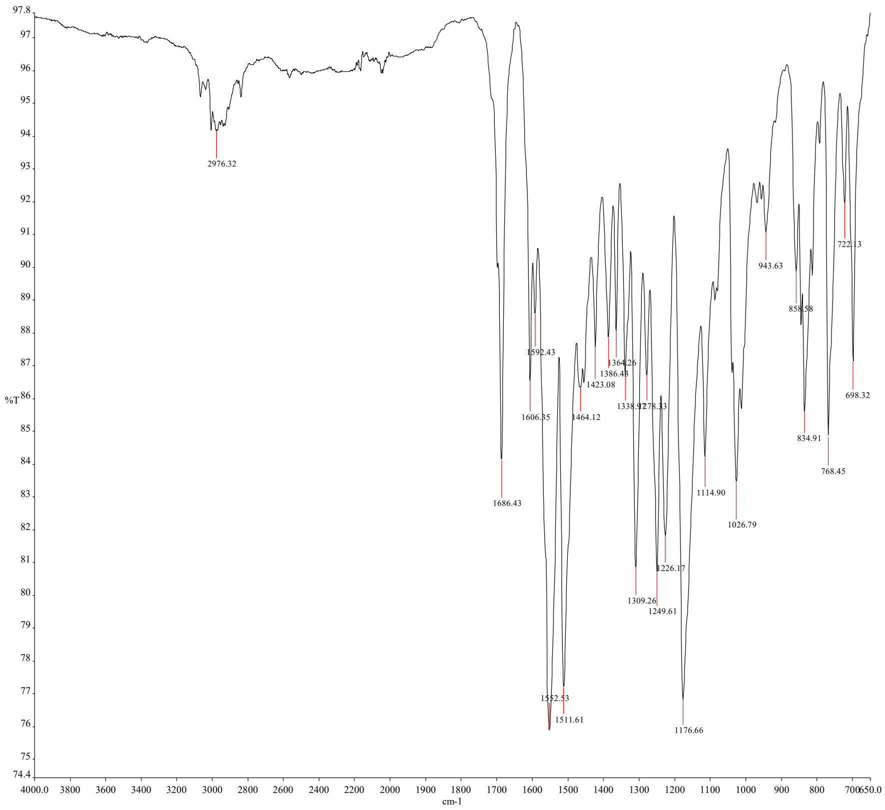

Fig. S28, ES‡

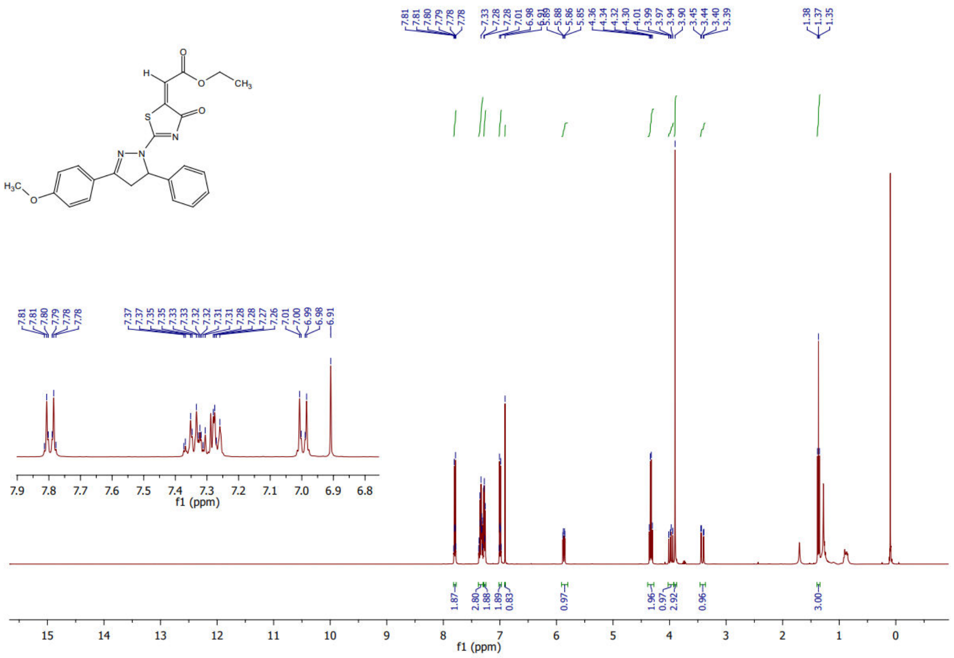

Fig. S29, ES‡

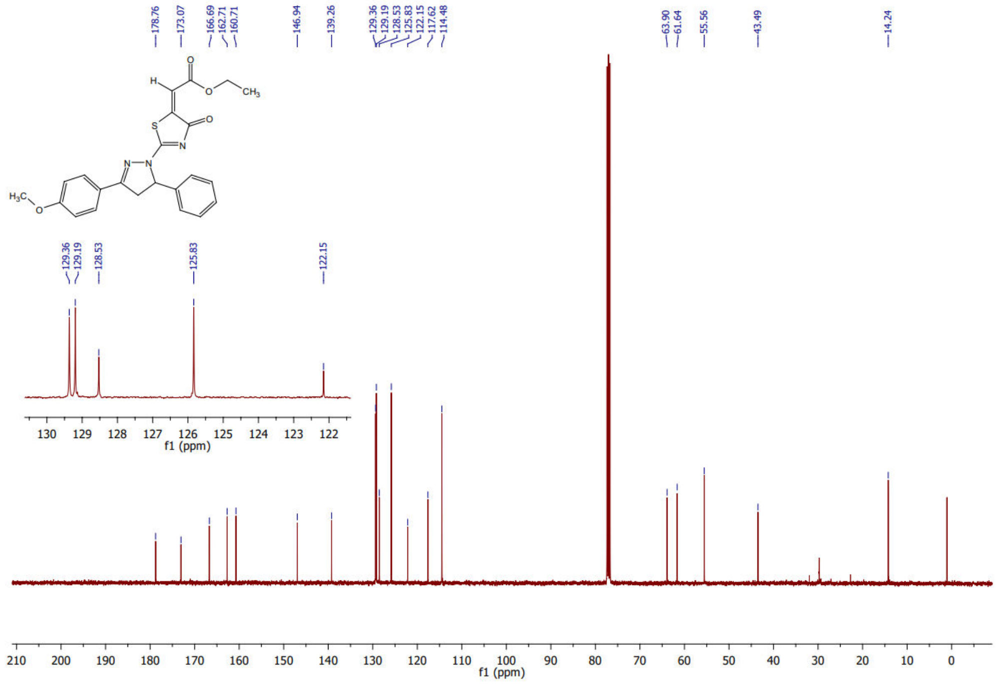

Fig. S30, ES‡

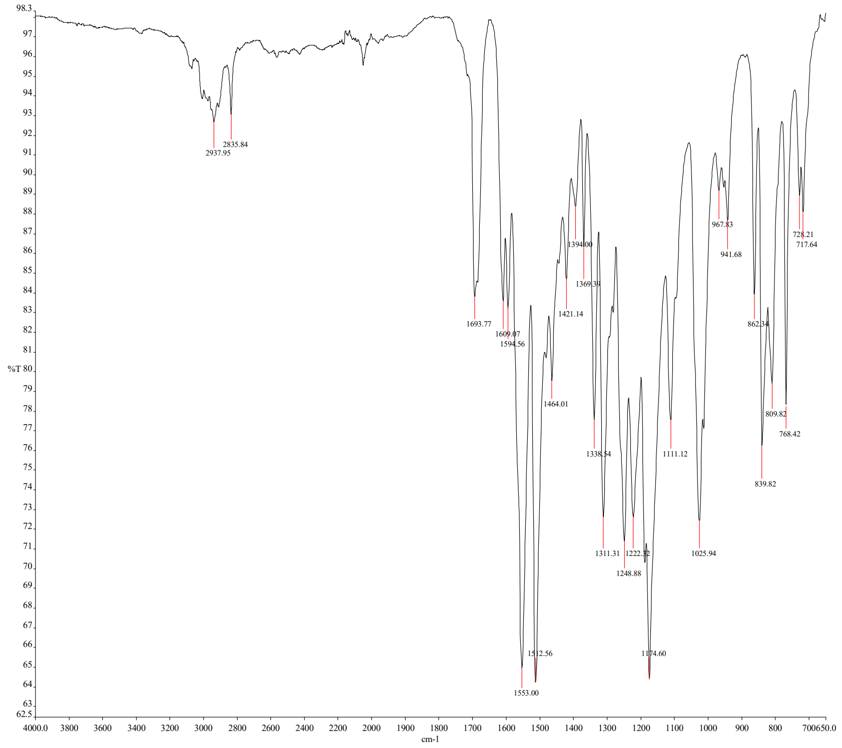

Fig. S31, ES‡

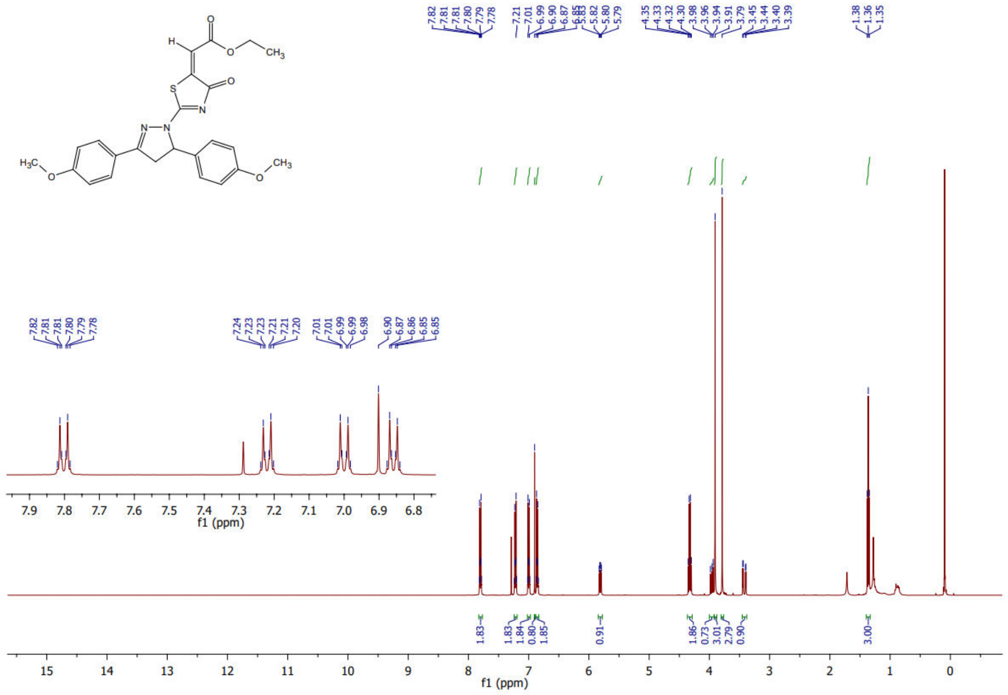

Fig. S32, ES‡

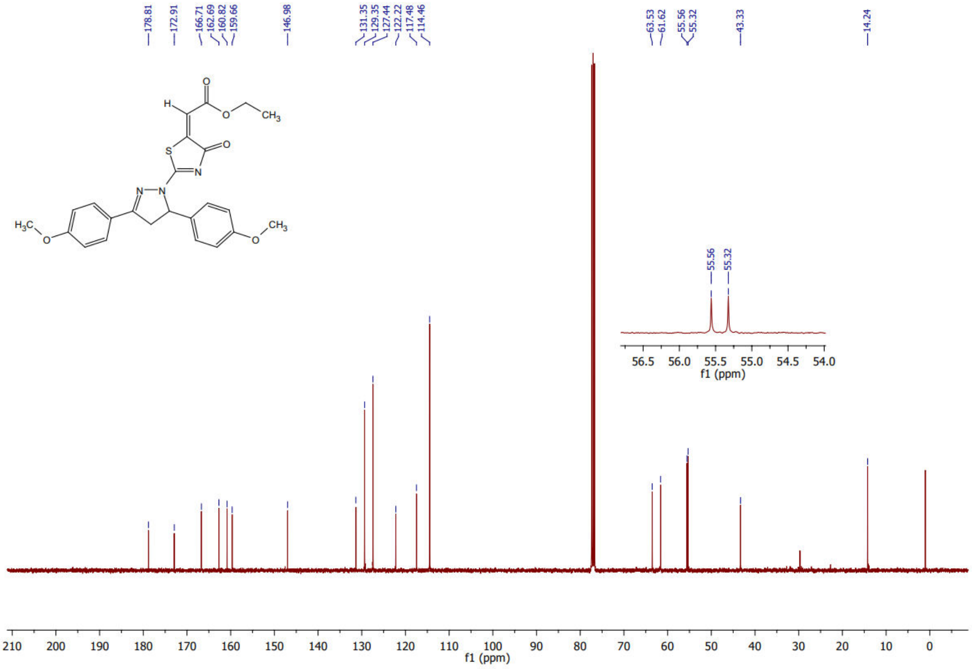

Fig. S33, ES‡

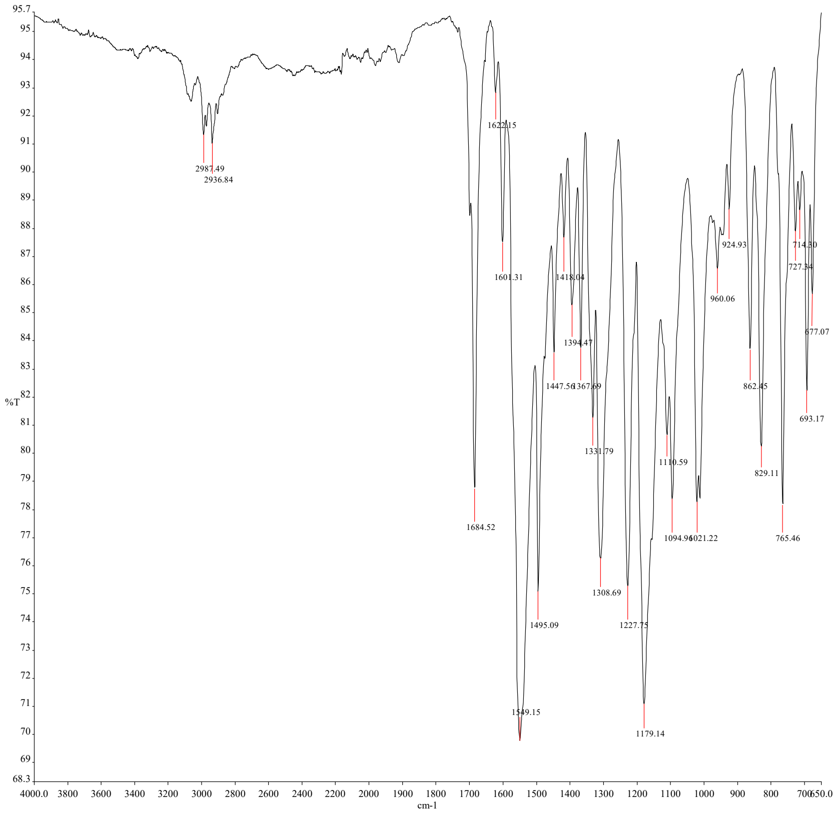

Fig. S34, ES‡

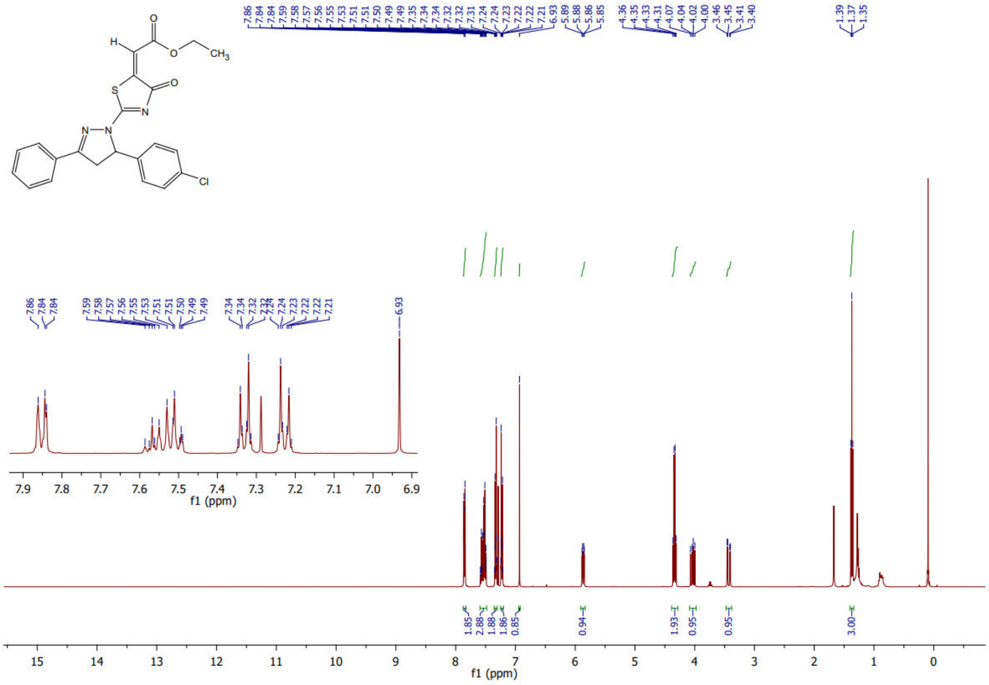

Fig. S35, ES‡

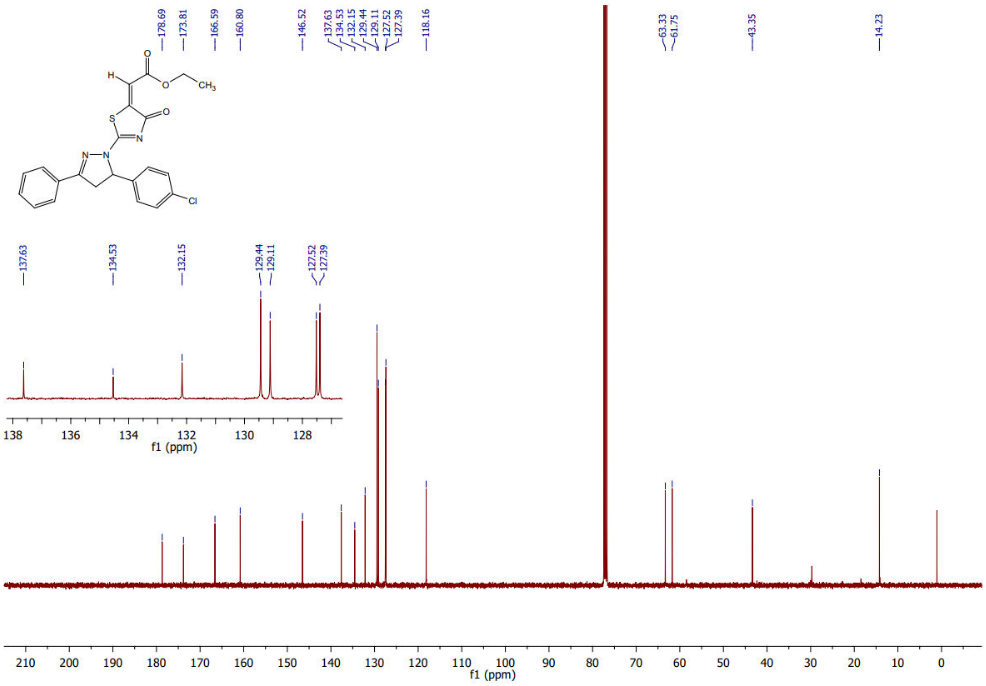

Fig. S36, ES‡

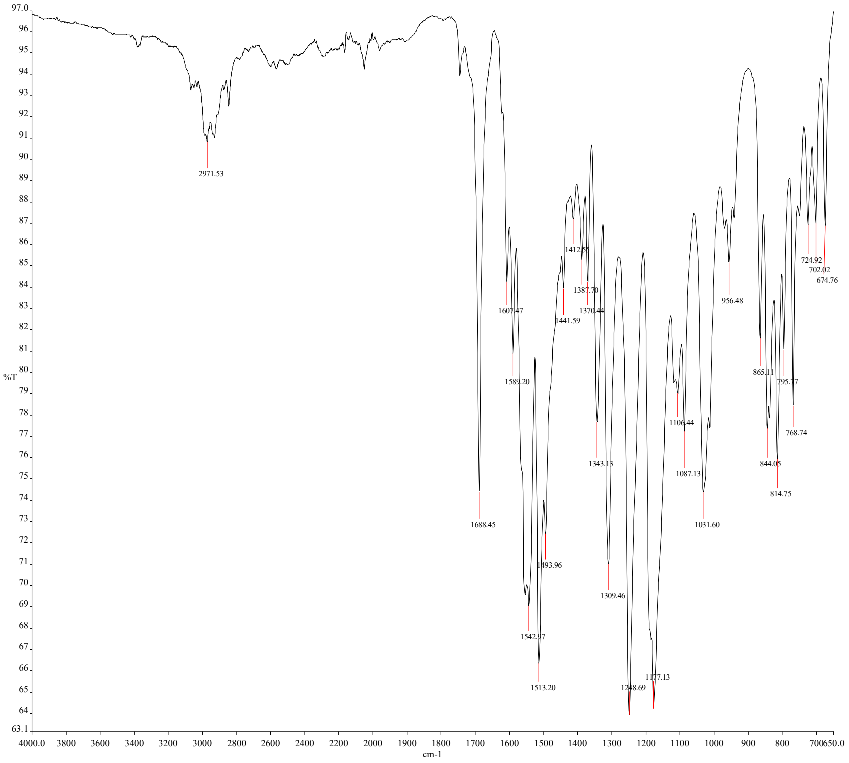

Fig. S37, ES‡

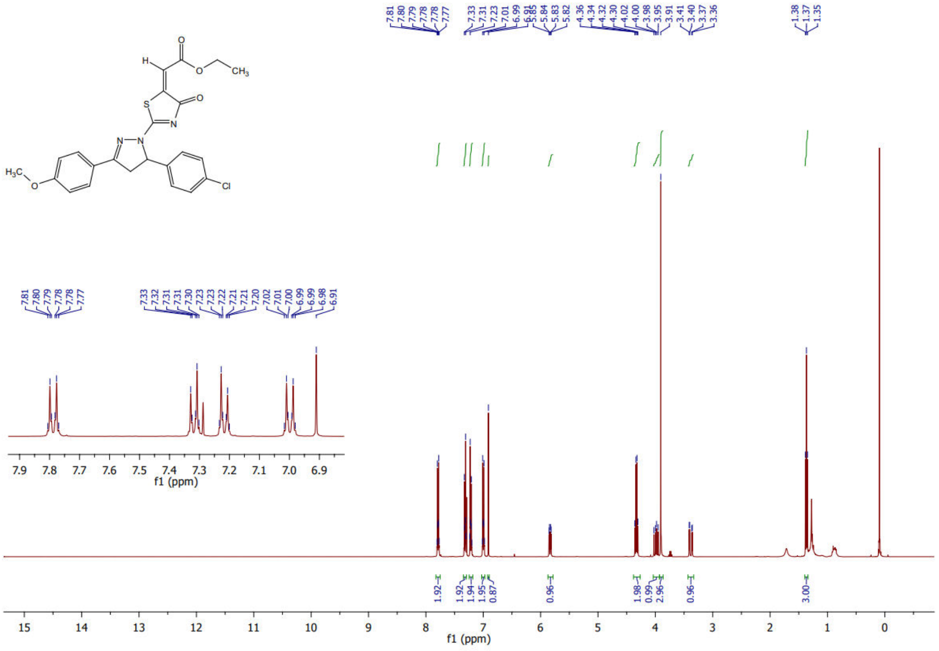

Fig. S38, ES‡

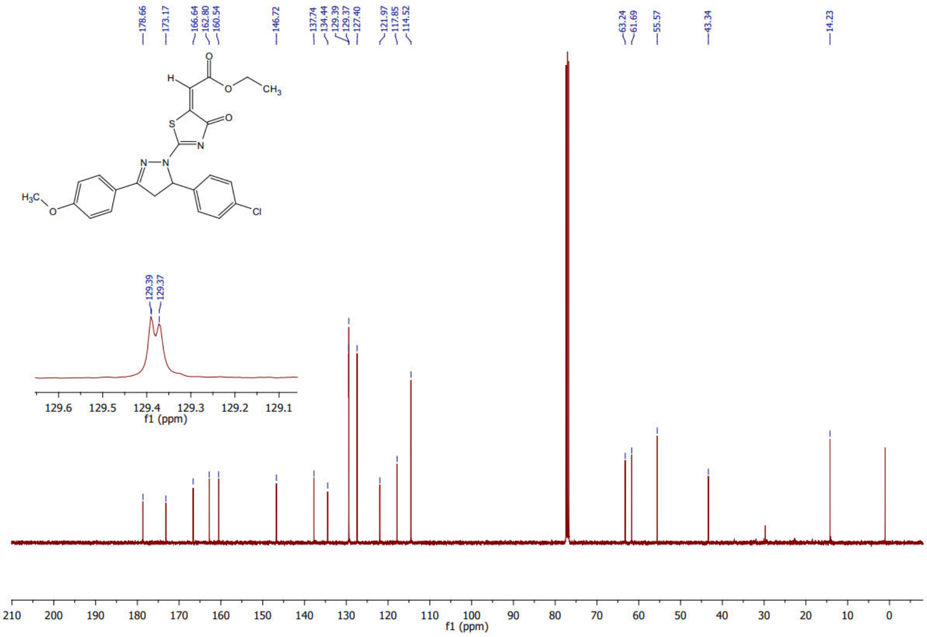

Fig. S39, ES‡

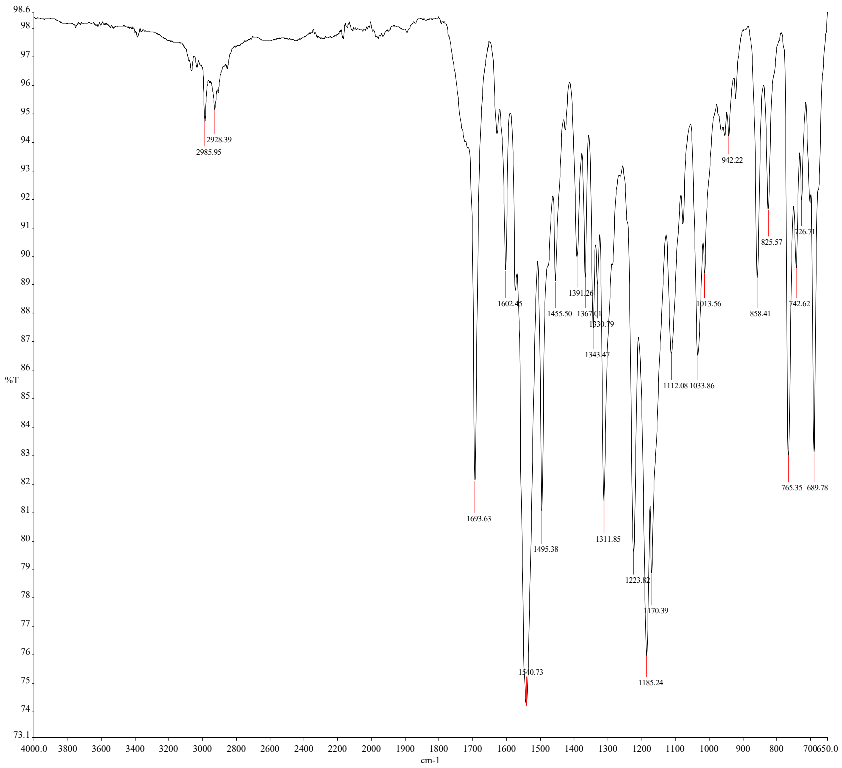

Fig. S40, ES‡

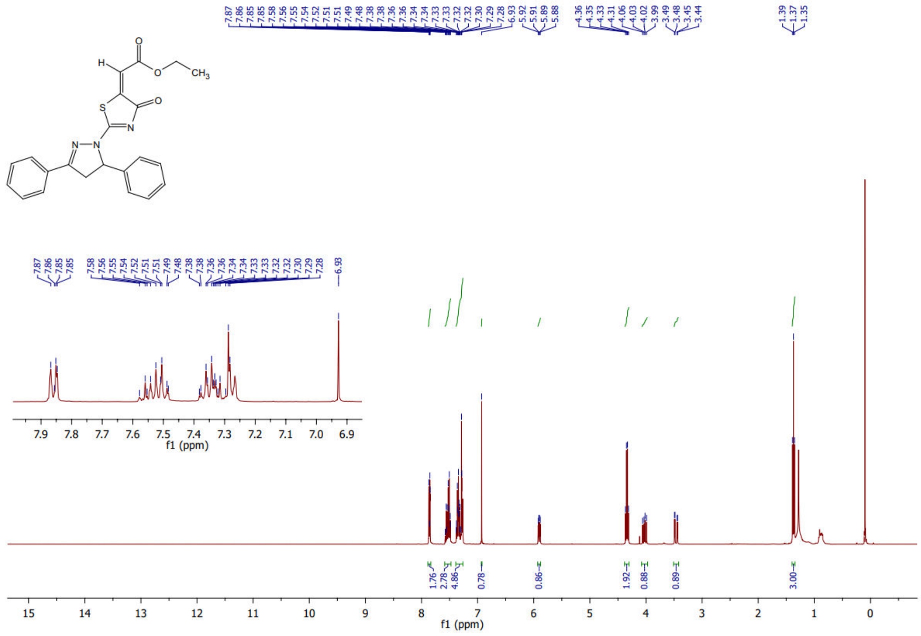

Fig. S41, ES‡

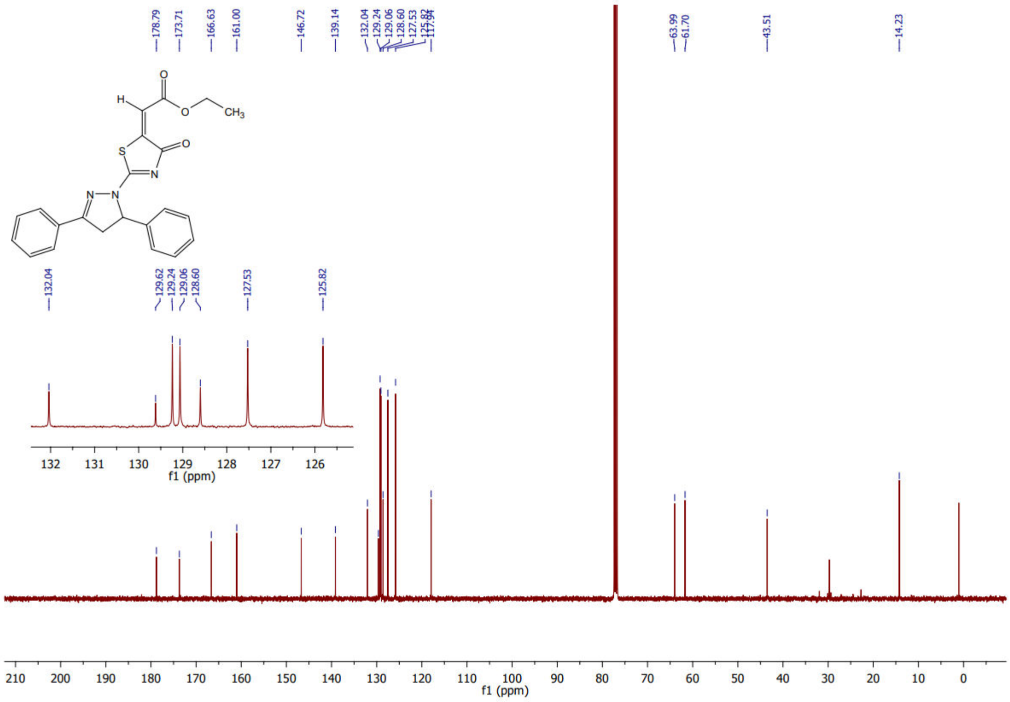

Fig. S42, ES‡

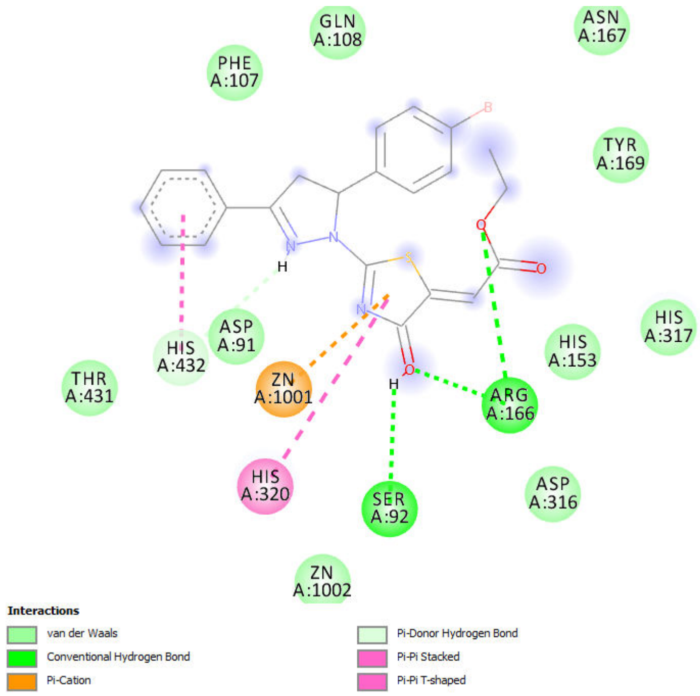

Fig. S43, ES‡

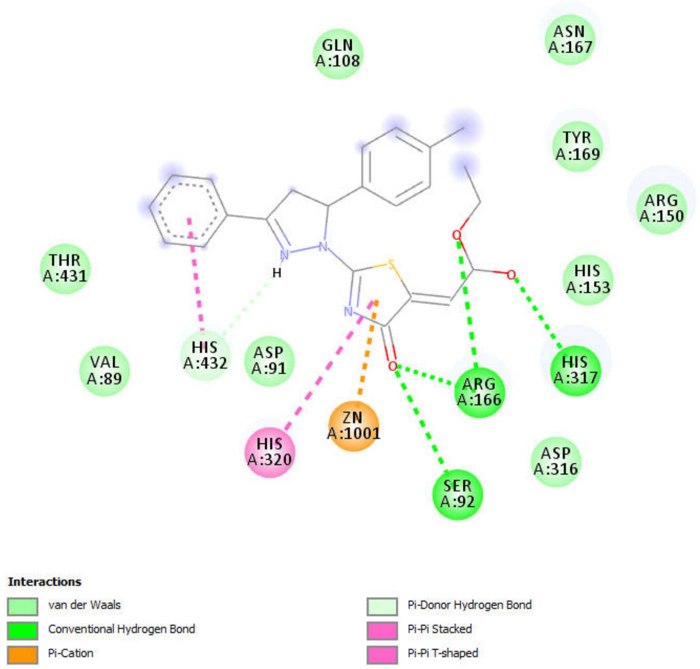

Fig. S44, ES‡

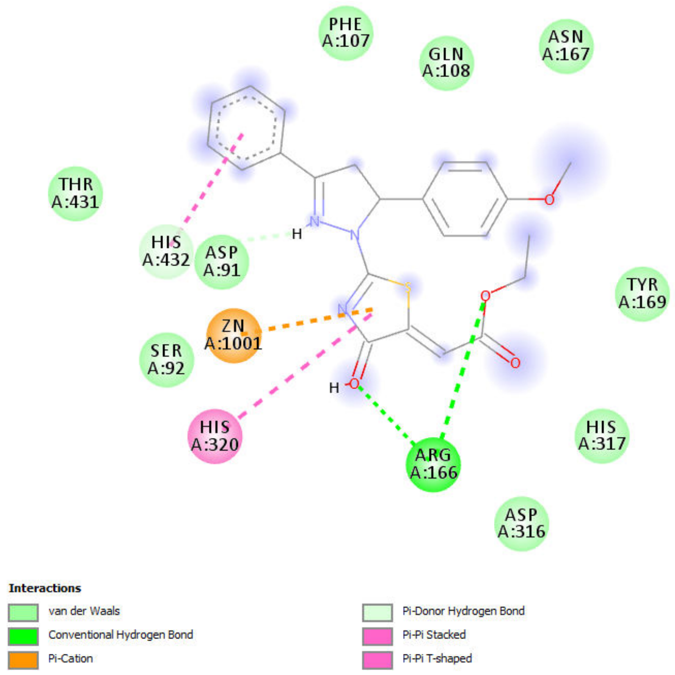

Fig. S45, ES‡

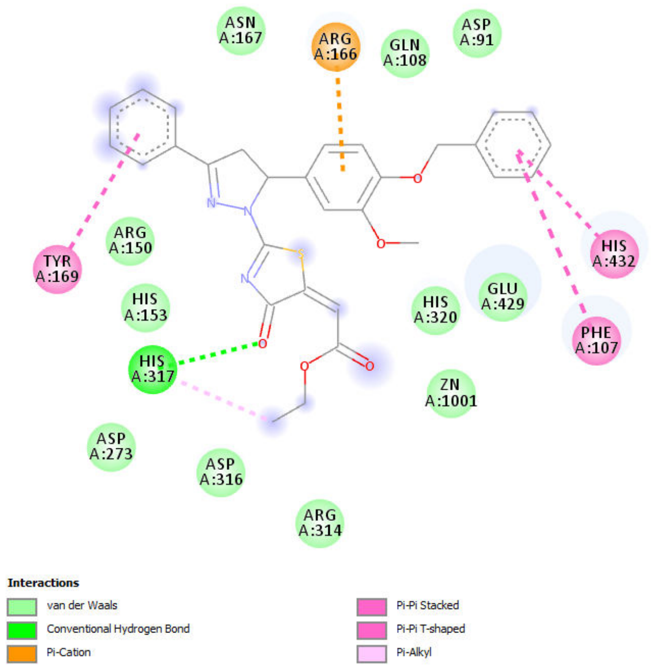

Fig. S46, ES‡

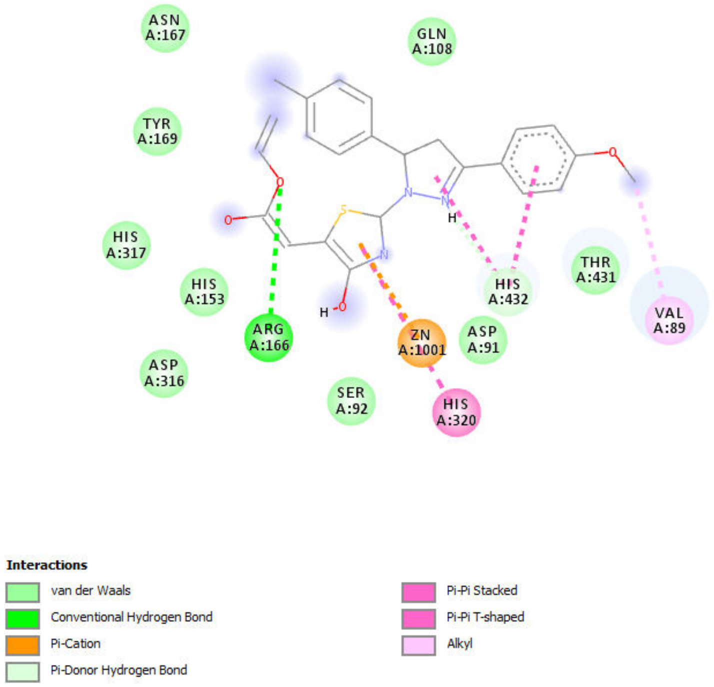

Fig. S47, ES‡

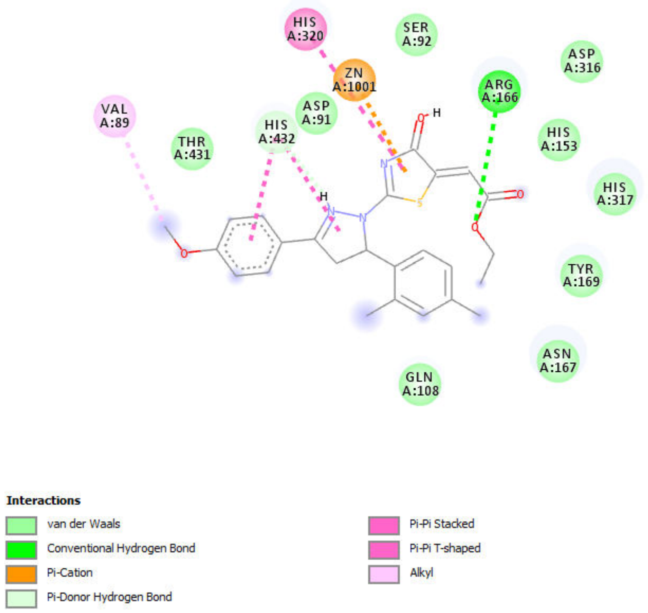

Fig. S48, ES‡

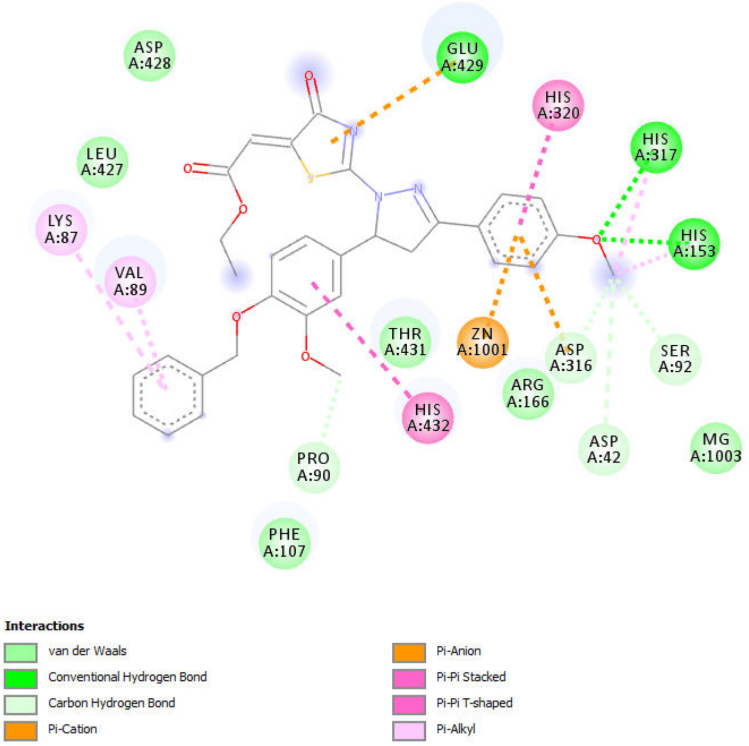

Fig. S49, ES‡

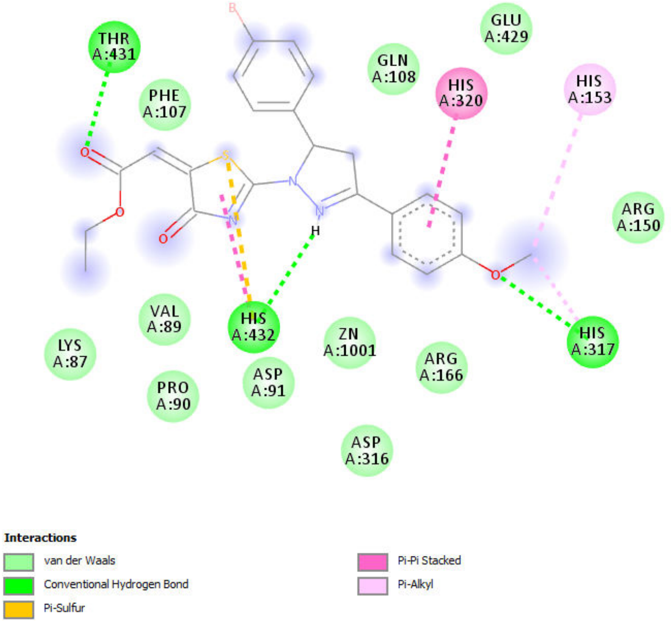

Fig. S50, ES‡

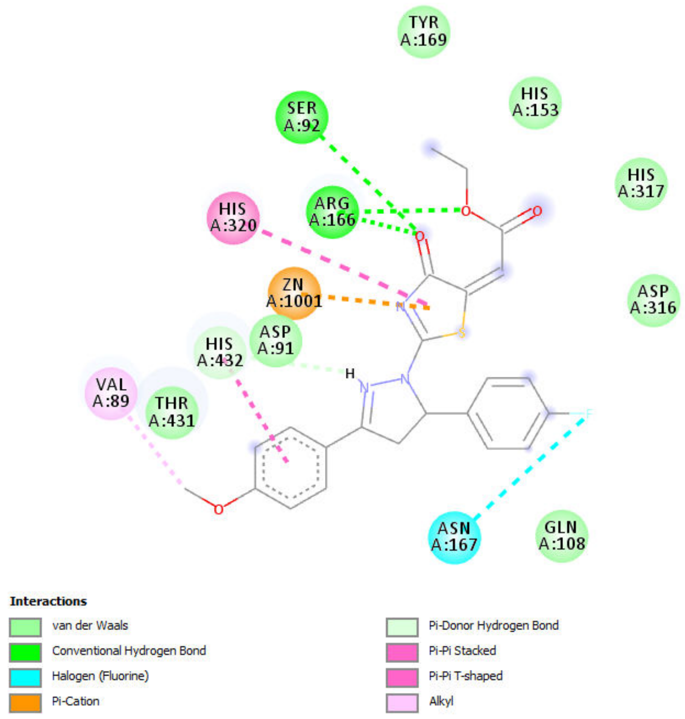

Fig. S51, ES‡

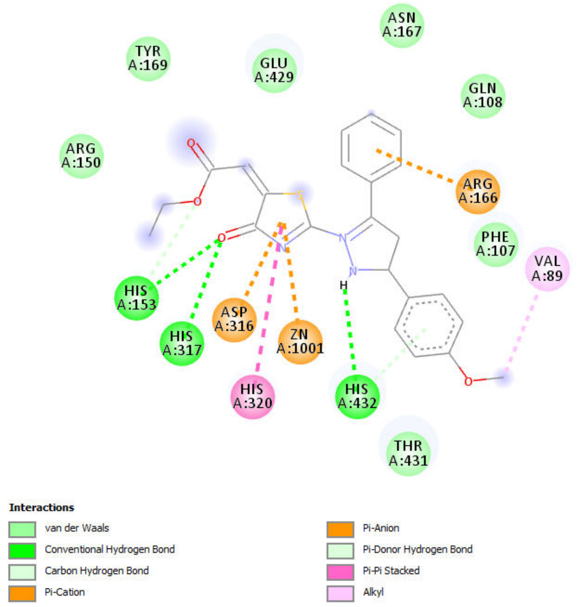

Fig. S52, ES‡

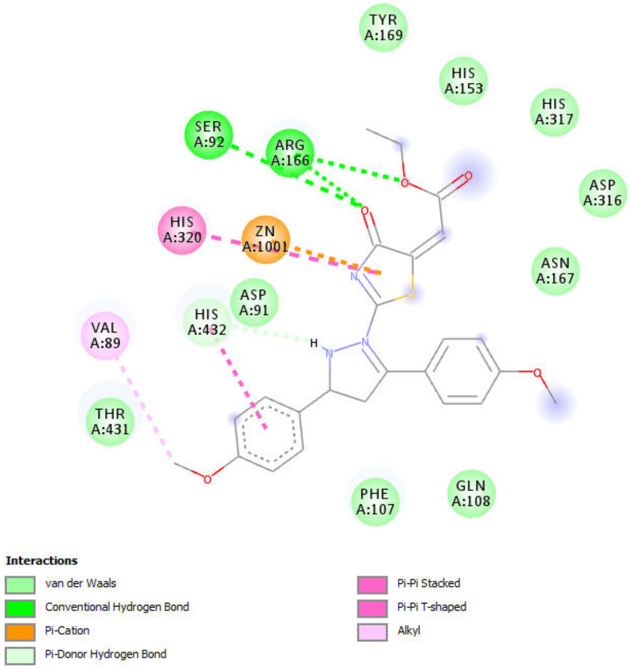

Fig. S53, ES‡

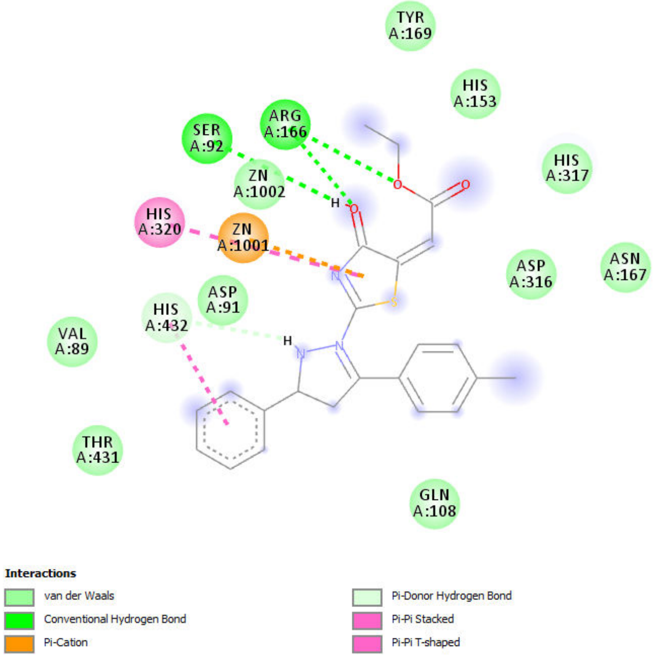

Fig. S54, ES‡

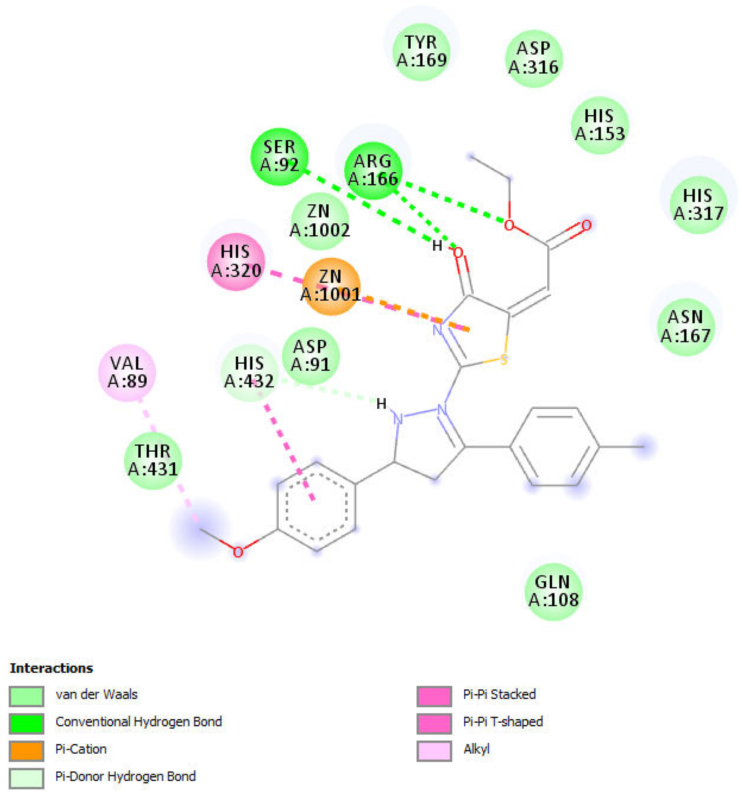

Fig. S55, ES‡

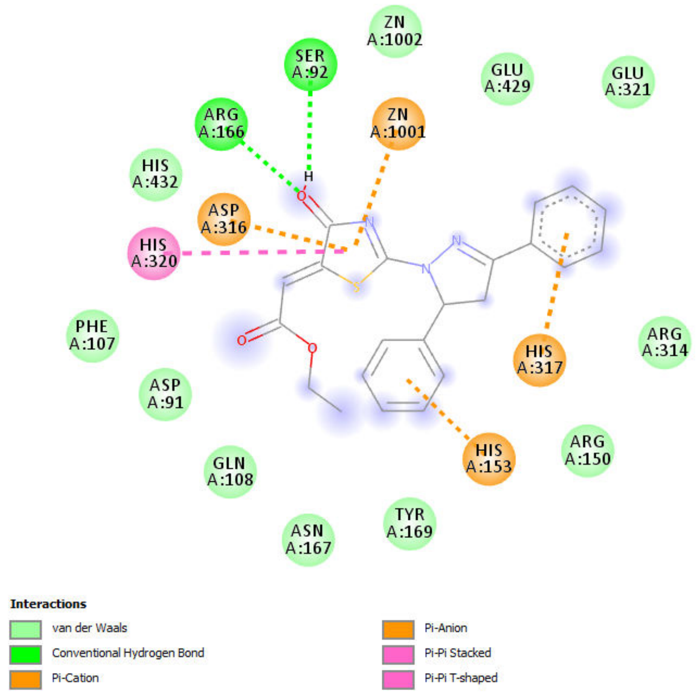

Fig. S56, ES‡

References:

1. Gottlieb HE, Kotlyar V, Nudelman A. NMR chemical shifts of common laboratory solvents as trace impurities. *J Org Chem*. 1997;62(21):7512-7515.
